# Gearing Up the Human Ankle-Foot System to Reduce Energy Cost of Fast Walking

**DOI:** 10.1038/s41598-020-65626-5

**Published:** 2020-05-29

**Authors:** Samuel F. Ray, Kota Z. Takahashi

**Affiliations:** Department of Biomechanics, University of Nebraska at Omaha, 6160 University Dr. South, Omaha, NE 68182 USA

**Keywords:** Bone quality and biomechanics, Musculoskeletal system, Muscle, Biomechanics, Physiology

## Abstract

During locomotion, the human ankle-foot system dynamically alters its gearing, or leverage of the ankle joint on the ground. Shifting ankle-foot gearing regulates speed of plantarflexor (i.e., calf muscle) contraction, which influences economy of force production. Here, we tested the hypothesis that manipulating ankle-foot gearing via stiff-insoled shoes will change the force-velocity operation of plantarflexor muscles and influence whole-body energy cost differently across walking speeds. We used *in vivo* ultrasound imaging to analyze fascicle contraction mechanics and whole-body energy expenditure across three walking speeds (1.25, 1.75, and 2.0 m/s) and three levels of foot stiffness. Stiff insoles increased leverage of the foot upon the ground  (p < 0.001), and increased dorsiflexion range-of-motion (p < 0.001). Furthermore, stiff insoles resulted in a 15.9% increase in average force output (p < 0.001) and 19.3% slower fascicle contraction speed (p = 0.002) of the major plantarflexor (Soleus) muscle, indicating a shift in its force-velocity operating region. Metabolically, the stiffest insoles increased energy cost by 9.6% at a typical walking speed (1.25 m/s, p = 0.026), but reduced energy cost by 7.1% at a fast speed (2.0 m/s, p = 0.040). Stiff insoles appear to add an extra gear unavailable to the human foot, which can enhance muscular performance in a specific locomotion task.

## Introduction

**Figure 1:**
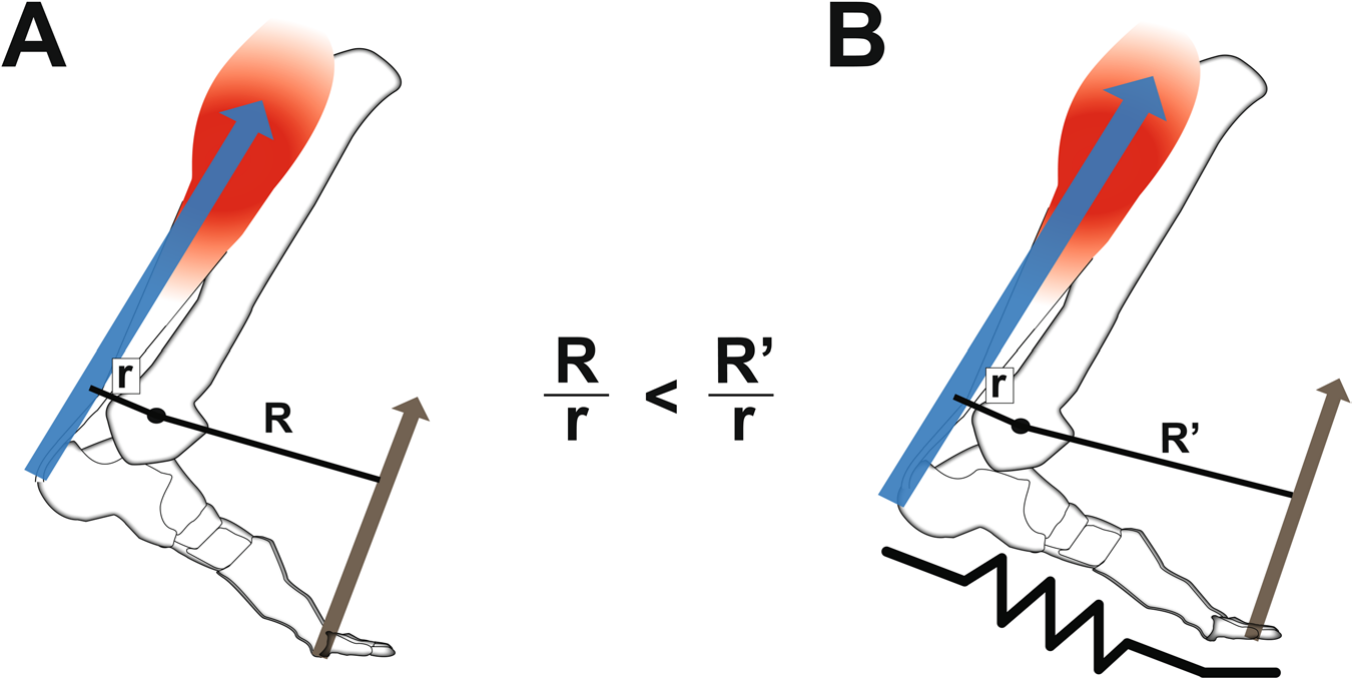
Effect of foot stiffness on ankle-foot gear ratio. (**A**) Gear ratio is the ratio of the lever arm of ground reaction force (R) and the lever arm of the ankle plantarflexor (calf) muscle (r) about the ankle joint center. (**B**) Lengthening the lever arm of ground reaction force (R’), by adding longitudinal foot stiffness, increases gear ratio and requires a greater force from the plantarflexor muscles to maintain a moment balance. Altering the gear ratio has been shown to shift force-velocity operating behavior of the plantarflexor muscles.

Humans take advantage of the functional interplay between the ankle joint and distal structures in the foot to walk and run effectively. Moreover, the plantarflexor muscle-tendon structures generate forces that help the body remain upright^1–3^ and move the body forward from one step to the next^4,5^. During walking or running, these muscles operate within favorable regions of force-length^6^ and force-velocity (i.e., near isometric or low speeds) relationships^7–9^ to produce force economically. Force production of the plantarflexors is facilitated by a gearing or lever-like function of the distal structures in the foot^10^. In particular, structures like the toes, arch, and intrinsic muscles^11^ can influence how the ground reaction force propagates underneath the foot, which in turn alters the force requirement of the ankle plantarflexor muscle-tendon unit. The ratio of the lever arms of the output ground reaction force and the input plantarflexor muscle-tendon force about the ankle^10^, termed *gear ratio*, can influence action of the plantarflexor muscles (Fig. 1). A high gear ratio can facilitate slower shortening of the plantarflexor muscles^12,13^, which could enhance force production, owing to the force-velocity relationship^14,15^. The ability to modulate ankle-foot gear ratio may then help to maintain optimal function in different locomotor tasks, including steady-state walking, running^8,16,17^, and maximal-acceleration push-off^10,16^.

Common, everyday devices like footwear can alter the gearing-like function of the foot, consequently altering locomotor performance^13,16,18^. For example, insole materials that minimize deflection around the toes can increase the lever arm of the ground reaction force, which in turn increases the gear ratio^13,19^. Such devices can lead to improved athletic performance, like faster sprinting^16^ and reduced metabolic cost of submaximal running^20,21^. It has been speculated that these performance benefits are derived from allowing major extensor muscles, like the ankle plantarflexors, to operate more effectively by modulating their force-velocity regions^19,21^.

A recent walking study involving *in vivo* muscle imaging data provided support for the theory that increasing the gear ratio (via shoes and insoles) directly affects the plantarflexor muscles’ force-velocity operating region^13^. In particular, increasing gear ratio increased plantarflexor (Soleus) muscular force output, while at the same time, decreased fascicle shortening velocity. However, this seemingly ‘favorable’ shift in force-velocity operation led to unchanged or increased whole-body metabolic energy cost at a normal walking speed (1.25 m/s)^13^. Such findings may be explained by potential competing effects of a disadvantageous increase in muscle force outweighing the beneficial decrease in fascicle shortening velocity at normal walking speeds. To elaborate, increases in plantarflexor force output (from added stiffness) might be expected to raise metabolic cost^22^, but decreases in ankle plantarflexor shortening velocity may unlock additional force-generating capacity of the muscle at a similar activation level. These competing effects potentially led to no metabolic benefit at a normal walking speed, likely because the plantarflexor muscles are already operating close to isometric, favoring economical force production^7–9,23^.

Studying locomotion modes in which plantarflexor muscles operate sub-optimally may be important to elucidate the role of gearing mechanisms of the foot and ankle. At fast walking speeds, for example, less ground contact time is available to propel the body forward, so a greater rate of force production is required, shifting plantarflexor muscles into faster and less economical operating conditions^17,23^ which likely increases metabolic cost. Both modeling and *in vivo* imaging data confirms that fast walking produces high plantarflexor shortening speeds^8,9,17,23^, which would have a detrimental effect on force output, requiring even greater muscle activation levels. While human foot muscles can stiffen the foot during walking^11^, it is possible that the foot muscles alone cannot stiffen enough to increase leverage needed for fast walking, possibly explaining why ankle joint work plateaus with increasing walking speed^24^. We theorized that adding stiff insoles could be a mechanism to “shift gears” for the ankle plantarflexors to a slower force-velocity operating region, which may be metabolically favorable during fast walking.

**Figure 2:**
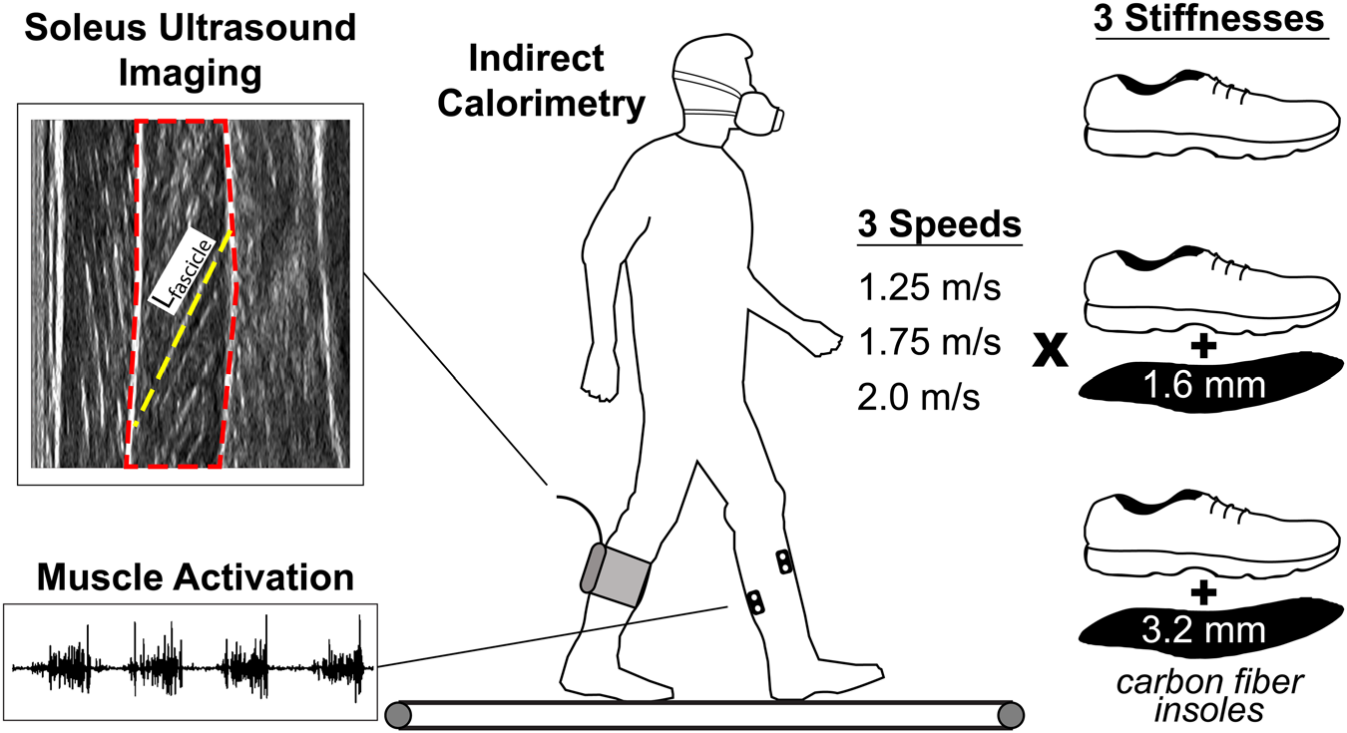
Experimental Setup. 15 young adults (12 males, 3 females, mean ± SD age 23 ± 2.1 yrs, height 176 ± 7.3 cm, mass 76.4 ± 12.4 kg) walked at a total of nine combinations of three walking speeds and three foot stiffness conditions. Kinematic measurements, kinetic data, muscle activation on the left leg, and soleus ultrasound muscle imaging on the right leg were recorded in one session, while metabolic gas measurements (indirect calorimetry) were recorded in a separate visit to the research site.

The purpose of our study was to determine if increasing gear ratio through added foot stiffness can reduce the metabolic cost of fast walking, and determine the effects of shifting plantarflexor force-velocity operating range on locomotion. We investigated subjects walking at three different speeds (1.25, 1.75, and 2.0 m/s) and three different foot stiffnesses (through combining shoes and carbon fiber insoles). We captured *in vivo* behavior of a major plantarflexor muscle (Soleus) through ultrasound imaging and electromyography (EMG), as well as lower extremity kinematics, kinetics, and whole-body metabolic energy expenditure (Fig. 2). We hypothesized that across all walking speeds, adding foot stiffness will reduce Soleus fascicle shortening velocity (i.e., slower contractions) and increase force output of the Soleus muscle (i.e., a shift in force-velocity operating region). Furthermore, we hypothesized that the effect of added foot stiffness on metabolic cost will be speed-dependent, in that added stiffness will be metabolically *detrimental* at normal walking speeds and metabolically *favorable* at fast speeds.

## Results

Three-point bending tests revealed average longitudinal bending stiffnesses of (mean ± s.d.) k = 7.2 ± 1.3 N/mm, 32.4 ± 8.7 N/mm, and 85.2 ± 25.9 N/mm for the three stiffness conditions: shoes alone, shoe + 1.6 mm insole, and shoe + 3.2 mm insole, subsequently referred to as *low*, *medium*, and *high* stiffness conditions, respectively.

**Figure 3:**
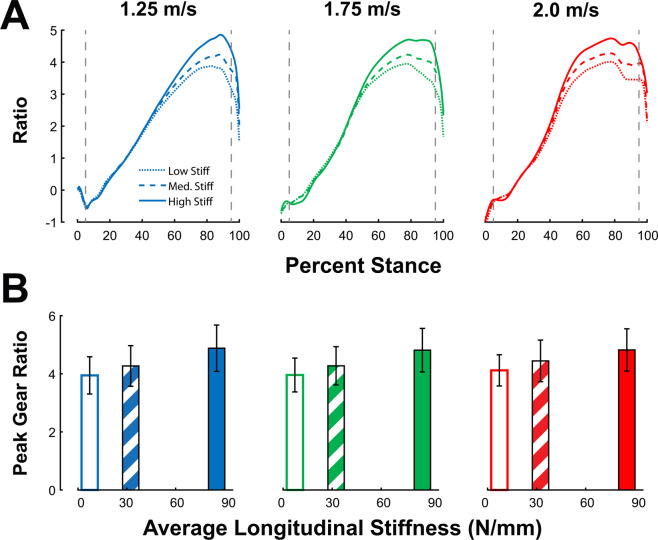
Gear ratio increases with speed and stiffness. Ankle-foot gear ratio are displayed across stance (**A**) as well as the peak (**B**) Peak gear ratio exhibited main effects of speed (p = 0.004) and stiffness conditions (p < 0.001). Peak gear ratio increased by 2.4% between slowest and fastest walking speeds, and increased by 20.8% between low and high foot stiffness conditions. Error bars signify s.d. between subjects.

Two-factor repeated measures ANOVA tests revealed a significant main effect of stiffness (p < 0.001) and speed (p < 0.001) on stance-averaged gear ratio, with no significant speed*stiffness interaction effect (p = 0.285). Peak gear ratio exhibited a significant main effect of speed (p = 0.004) and stiffness (p < 0.001), with a significant speed*stiffness interaction effect(p = 0.028). The effect of stiffness had a greater influence on gear ratio than walk speed; we observed a 16.2% increase in average gear ratio and a 20.8% increase in peak ratio between *low* to *high* foot stiffness conditions, and a smaller 8.1% and 2.4% increase in average and peak gear ratio between 1.25 to 2.0 m/s walking speeds, respectively (Fig. 3).

**Figure 4:**
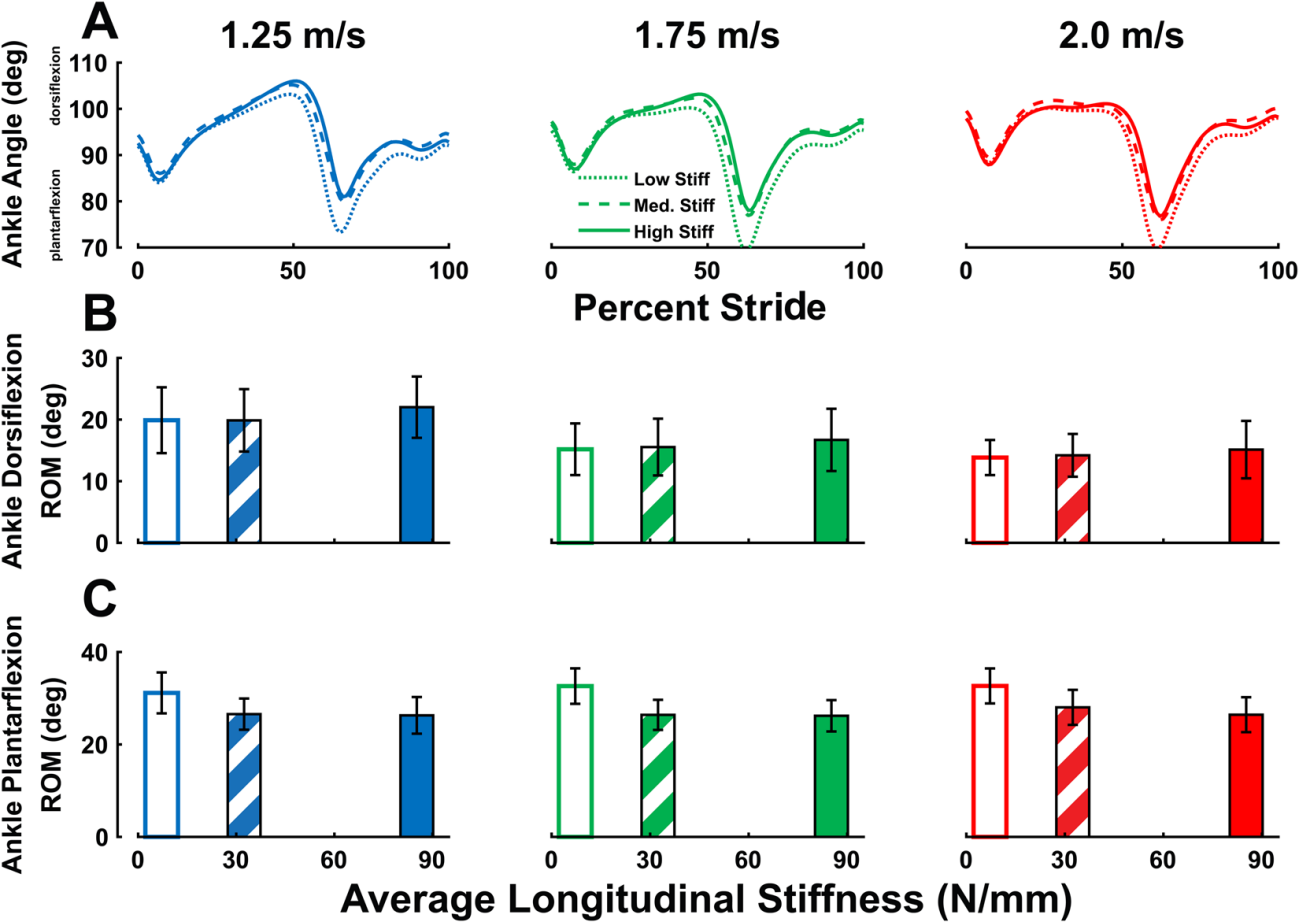
Ankle range-of-motion is altered with speed and stiffness. (**A**) Ankle angle range of motion (ROM) is displayed across stride. (**B**) Ankle dorsiflexion ROM exhibited main effects of speed (p < 0.001) and stiffness (p < 0.001), increasing by an average of 10% between *low* and *high* stiffness conditions. (**C**) Ankle plantarflexion ROM did not exhibit a significant effect of walking speed (p = 0.301) but did show an effect of foot stiffness (p < 0.001), decreasing by an average of 18.1% from *low* to *high* stiffness conditions. Error bars signify s.d. between subjects.

Ankle range-of-motion (ROM) was altered in two ways by stiff insoles. Ankle dorsiflexion ROM showed a significant main effect of speed (p < 0.001) and stiffness (p < 0.001) with no significant interaction effect (p = 0.462), and we observed a 10.0% increase between *low* and *high* foot stiffness conditions, and a 30.1% decrease between 1.25 and 2.0 m/s (Fig. 4). Ankle plantarflexion ROM showed a significant main effect of stiffness (p < 0.001) but not speed (p = 0.301), with no significant interaction effect (p = 0.133), and we observed a 18.1% decrease from *low* to *high* stiffness conditions.

**Figure 5:**
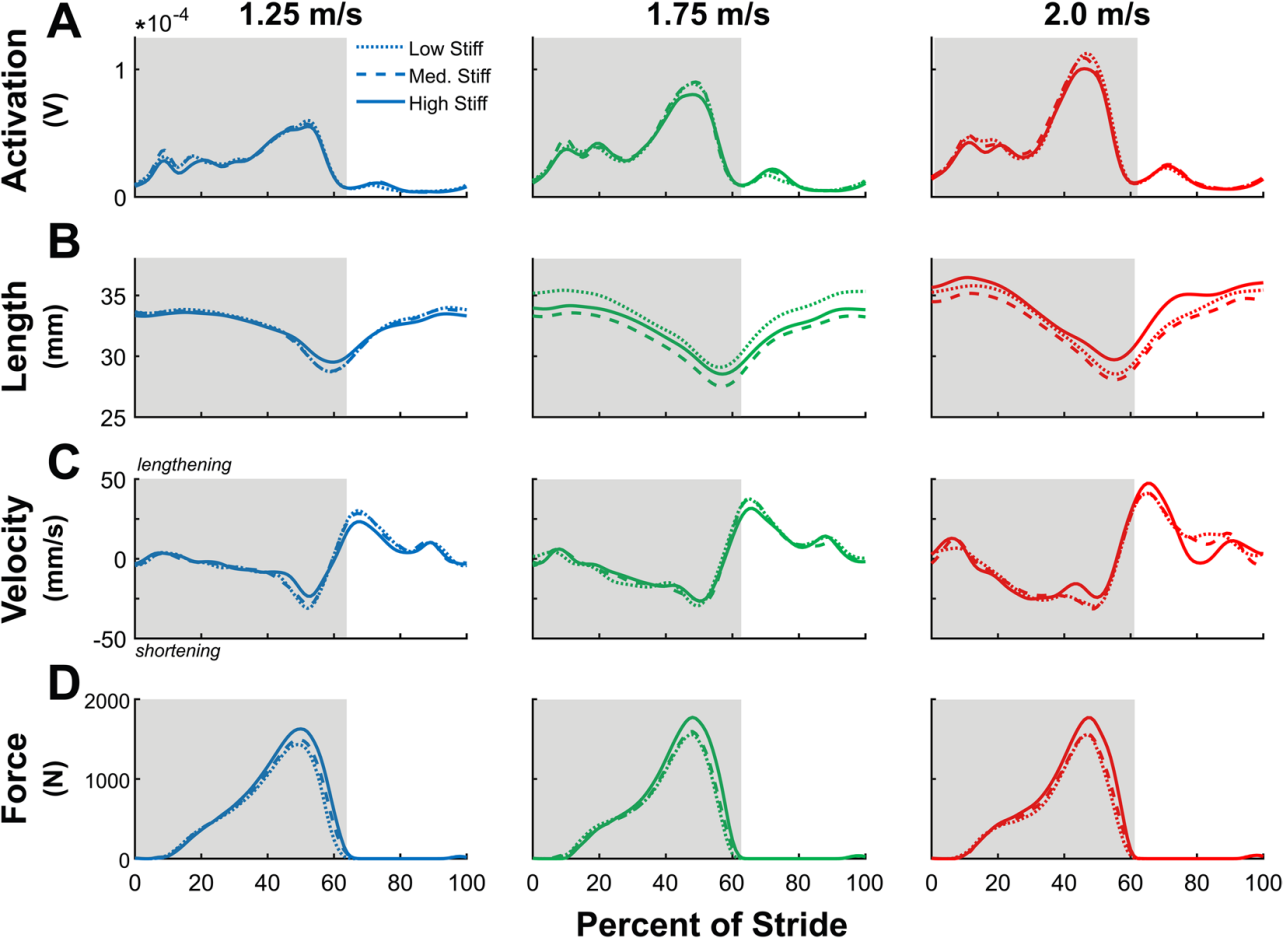
Soleus muscle time-series data. Stride-normalized data of (**A**) Soleus activation (N = 14, left limb), (**B**) Soleus fascicle length (N = 15, right limb), (**C**) Soleus fascicle velocity, and (**D**) Soleus fascicle force across different walk speeds. Stance-phase data is highlighted in grey. Soleus fascicle shortening velocity decreased with added stiffness and increased with faster walk speeds, and fascicle force increased with both added stiffness and faster walking speeds. Soleus EMG activation increased with faster walk speeds, but did not change with added foot stiffness.

**Figure 6:**
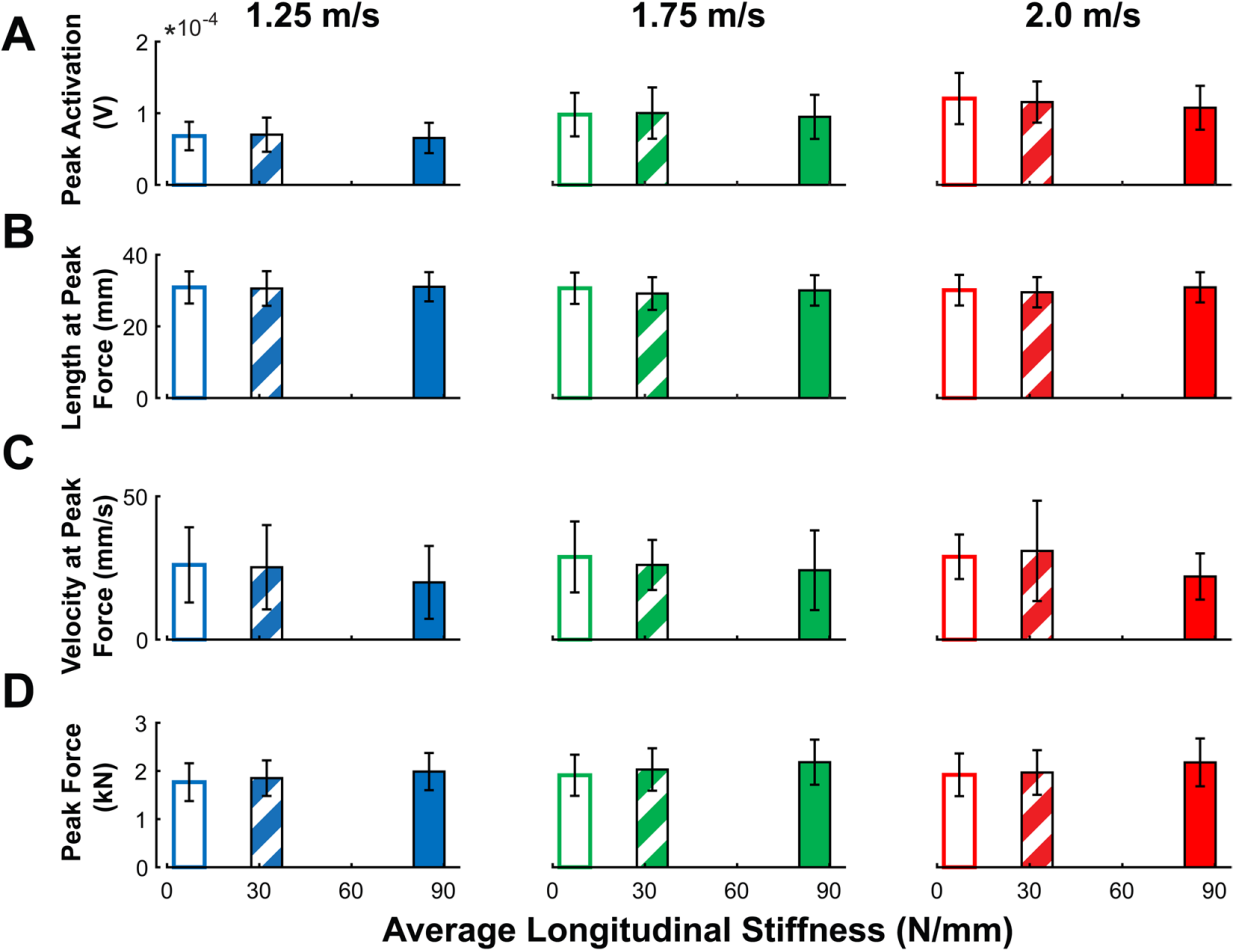
Soleus muscle group mean data. (**A**) Peak soleus activation increased with walking speed (p < 0.001) but did not change with added stiffness (p = 0.060). (**B**) Fascicle length at time of peak force generation exhibited a small increase with added foot stiffness (p = 0.034) but did not change with faster walk speeds (p = 0.190). (**C**) Fascicle shortening velocity at time of peak force increased with walking speed (p = 0.038) and decreased with added foot stiffness (p = 0.014). (**D**) Peak fascicle force significantly increased with walking speed (p < 0.001) and stiffness (p < 0.001). Error bars signify s.d. between subjects.

We observed a significant positive main effect of speed on stance-averaged Soleus fascicle shortening velocity (p < 0.001), and from 1.25 to 2.0 m/s walking speeds, average shortening velocity increased by 78.4% (Supplementary Fig. 12). Meanwhile, there was a significant negative effect of stiffness on average shortening velocity (p = 0.002), with an approximate 19.3% decrease between *low* and *high* stiffness conditions (Supplementary Fig. 12). Fascicle shortening velocity at time of peak force exhibited a main effect of foot stiffness (p = 0.014) (Figs. 5 and 6) and walk speed (p = 0.038), decreased by an average of 21.1% between *low* and *high* stiffness conditions, and increased 14.6% from 1.25 to 2.0 m/s. Average Soleus fascicle length did not exhibit main effects of foot stiffness (p = 0.067) or speed (p = 0.182) (Supplementary Fig. 12). Fascicle length at time of peak force had no main effect of walking speed (p = 0.190) but did have an effect of foot stiffness (p = 0.034), exhibiting a small 0.42% increase from *low* to *high* stiffness conditions. No significant speed*stiffness interaction effects were observed for average shortening velocity (p = 0.652), velocity at time of peak force (p = 0.550), average fascicle length (p = 0.255), or length at peak force (p = 0.370).

Integrated Soleus EMG activation had significant effects of walking speed (p < 0.001), increasing by 23.5% between 1.25 and 2.0 m/s, but had no effects from changes in stiffness (p = 0.145) and no interaction effect (p = 0.635). Peak Soleus EMG activation as well had a significant effect of walking speed (p < 0.001), increasing by 68.8% between 1.25 and 2.0 m/s, but had no effect from stiffness (p = 0.060) or interaction (p = 0.156) (Figs. 5 and 6). Lastly, average stance-phase Soleus fascicle force did not change with increasing walking speed (p = 0.269) but did have a main effect with stiffness (p < 0.001), increasing by an average of 15.9% between low and high stiffness conditions, as well as not displaying an interaction effect (p = 0.266). Peak Soleus fascicle force had significant effects of walking speed (p < 0.001) and stiffness (p < 0.001), increasing approximately 8.2% between 1.25 and 2.0 m/s and increasing 13.4% between *low* and *high* stiffness conditions.

**Figure 7:**
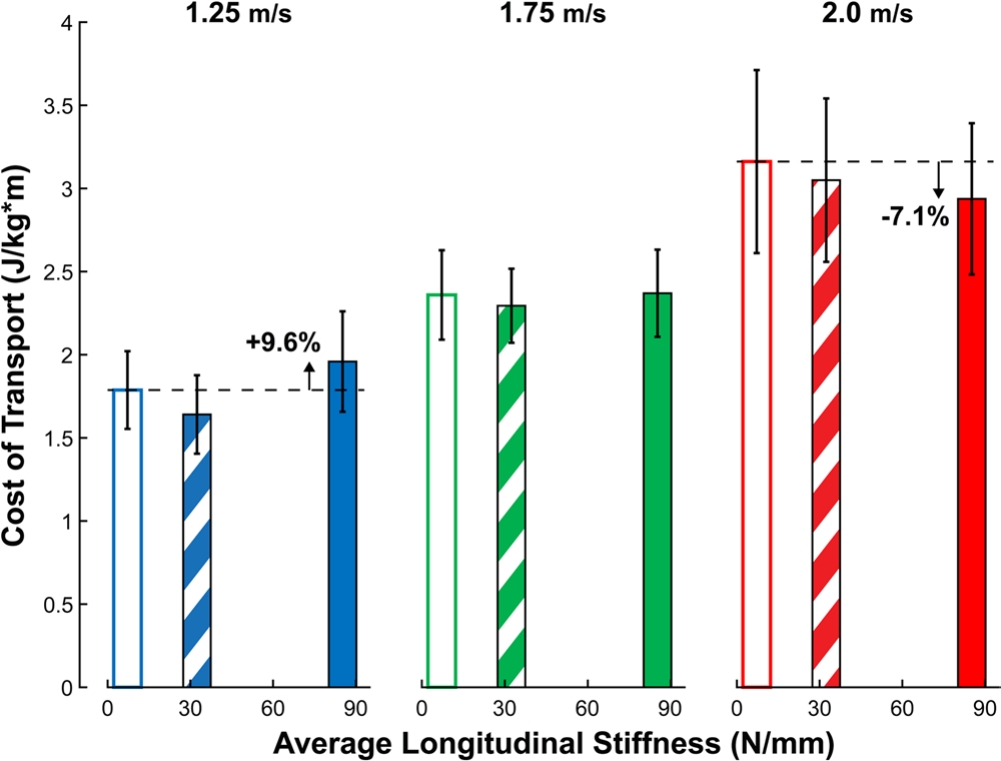
Stiff insoles have speed-dependent effects on metabolic cost. Whole-body metabolic cost (N = 15, mean ± SD) exhibited a significant main effect of speed (p < 0.001) but not stiffness (p = 0.064), as well as a significant speed*stiffness interaction (p < 0.001). Post-hoc comparisons found there were significant differences between the *low* vs *high* stiffness (p = 0.026) and *medium* vs *high* stiffness (p = 0.015) conditions at 1.25 m/s. Also, at 2.0 m/s, we found a difference between the *low* vs *high* stiffness (p = 0.040) conditions. At 1.25 m/s, the stiffest condition (K = 85.2 N/mm) increased metabolic cost by 9.6% compared to the lowest stiffness condition (K = 7.2 N/mm). Meanwhile, at 2.0 m/s, the same stiffness reduced whole-body metabolic cost by 7.1%. Error bars signify s.d. between subjects.

There was a significant main effect of walking speed (p < 0.001), no main effects of foot stiffness, (p = 0.064), and a significant speed*stiffness interaction effect on whole-body metabolic cost of transport (p < 0.001) (Fig. 7). We observed a group mean increase of 70.9% in metabolic cost from 1.25 to 2.0 m/s walking speed, and speed-dependent effects of foot stiffness. Post-hoc comparisons performed between stiffness conditions at each speed using Fisher’s Least Significant Difference adjustment found there were significant differences between the *low* vs *high* stiffness (p = 0.026) and *medium* vs *high* stiffness (p = 0.015) conditions at 1.25 m/s. Also, at 2.0 m/s, post-hoc comparisons revealed a difference between the *low* vs *high* stiffness (p = 0.040) conditions. At 1.25 m/s walking, the *high* stiffness condition resulted in a 9.6% increase in metabolic cost compared to *low* stiffness, while the same *high* stiffness condition resulted in a 7.1% decrease in metabolic cost at 2.0 m/s walking.

## Discussion

**Figure 8:**
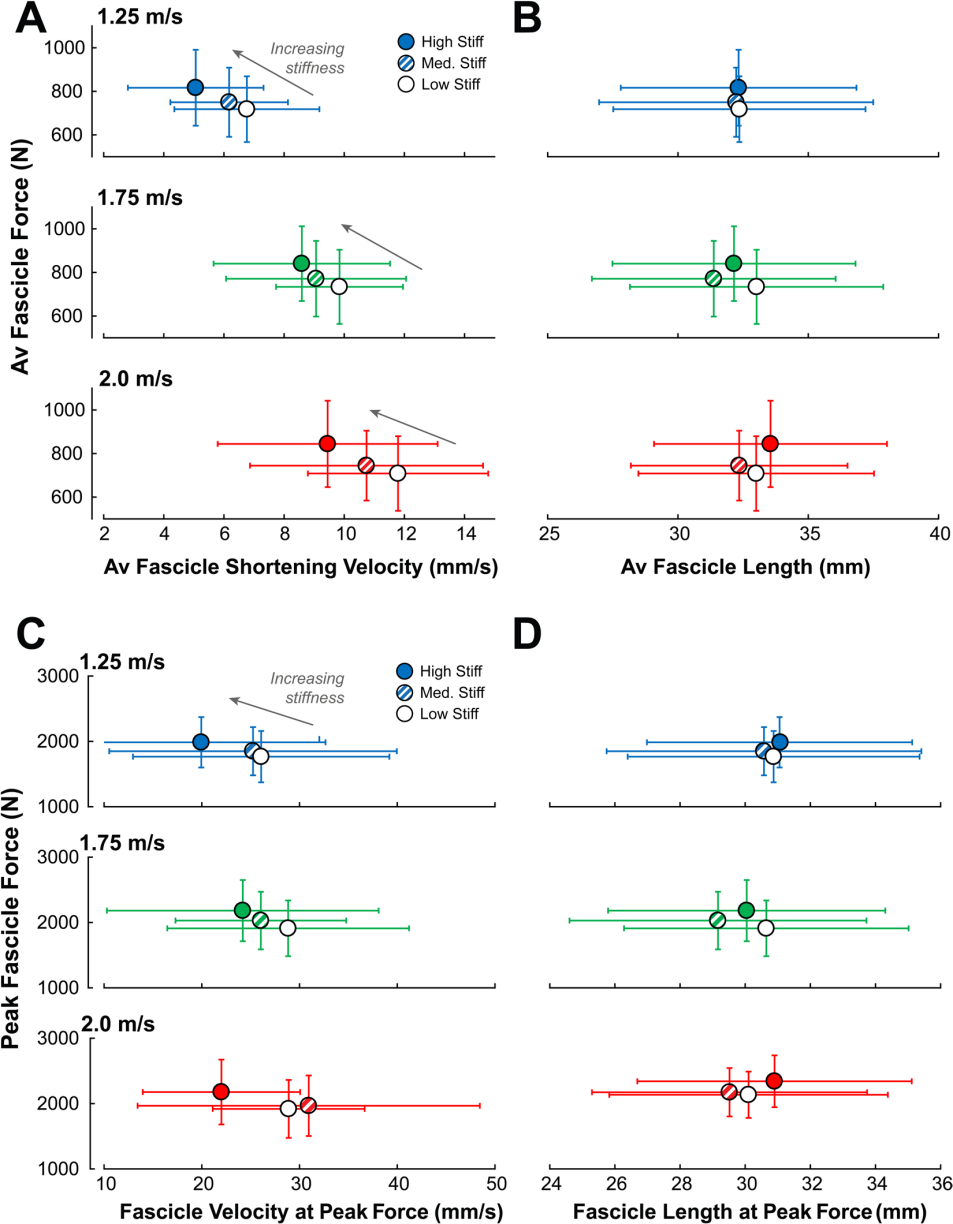
Added foot stiffness shifts Soleus muscle Force-velocity operating region. Stance-averaged Soleus fascicle force data were plotted as a function of: (**A**) average fascicle shortening velocity during stance and (**B**) average fascicle length during stance. The peak Soleus fascicle force data were also plotted as a function of: (**C**) fascicle shortening velocity at the time of peak force, and (**D**) fascicle length at the time of peak force. Added foot stiffness was able to increase Soleus force output without added muscle activation (Fig. 6 and Supplementary Fig. 10), suggesting that the increased force was primarily due to a shift in the force-velocity operating region. Error bars signify s.d. between subjects.

The purpose of this study was to determine how manipulating ankle-foot gearing through stiff insoles would affect whole-body metabolic energy cost across a range of walking speeds. The stiff insoles used in this study increased peak gear ratio by roughly 20.8%, which also increased ankle dorsiflexion and decreased plantarflexion motion during stance. We hypothesized that stiff insoles would decrease the shortening speed of Soleus muscle fascicles, increase the force output of the Soleus, and would have a speed-dependent effect on whole-body metabolic energy cost. All three of our hypotheses were supported, as stiff insoles decreased Soleus contraction speed and increased force output and led to both a 9.6% increase in metabolic cost at 1.25 m/s and a 7.1% metabolic cost reduction when walking at 2.0 m/s. Our experimental results add evidence to the theory that simple passive devices (i.e., stiff insoles) that can alter the ankle-foot gear ratio can shift force-velocity operating region of the Soleus muscle, by increasing force output and decreasing shortening velocity of muscle fascicles. The shift in muscle operation is most evident from the increases in force generated by the Soleus muscle fascicles (Fig. 8), with a small change in operating length (Fig. 8) and notably without a corresponding increase in EMG activation (Fig. 6). This force-velocity shift is in agreement with a prior study^13^ that observed an increase in Soleus force per unit EMG activation when walking with added foot stiffness. At 1.25 m/s walking speeds, the increased force output from stiff insoles likely drove up metabolic cost of walking, but had an inverse effect at the fastest speed (2.0 m/s).

The shifting of the force-velocity region of the Soleus muscle possibly explains the reduction of metabolic cost at the fastest walking speed. At 2.0 m/s walking, the stiff insole reduced average stance-phase shortening velocity from 11.8 mm/s to 9.5 mm/s, and velocity at the time of peak force from 28.9 mm/s to 22.0 mm/s. It is possible that the stiff insoles restored some function to the plantarflexors that had been lost at faster walk speeds, which could explain the speed-dependent effect on metabolic cost. At normal walking speeds, plantarflexors produce force with low shortening velocity, especially through mid-stance^7^, favoring economical force production. Our study also corroborates an increase in concentric contraction with faster walking speeds^8,17,23^, evident from the increases in average shortening velocity during entire stance and velocity at the time of peak force production. Stiff insoles were able to restore fascicle contractions closer to isometric levels, possibly restoring economical force production. However, at normal walking speeds, this shift in force-velocity behavior may not be required by the Soleus muscles, and as such, an increase in force production is not necessary, and increases overall metabolic cost of walking. Stiff insoles also reduced the amount of ankle joint positive work performed over stance (Supplementary Fig. 5), even though insoles increased both average force output of the Soleus muscles (Fig. 6) and the ankle plantarflexion moment (Supplementary Figs. 2 and 3). Following this, decreases in work performed at the ankle could be outweighed by increases in force production at normal walking speeds. When walking at fast speeds, increase in force production economy could outweigh the increases in force production. It appears that these insoles are able to add an extra “gear” to the foot, corroborated by our results on average and peak gear ratio (Fig. 3). Peak gear ratio increased by 4.4% between 1.25 and 2.0 m/s walking without insoles, while the stiffest insoles increased peak gear ratio themselves by an average of 20.8%. This extra gear may not be attainable through biological foot structures alone, perhaps due to small cross-sectional area of foot muscles^11^. Thus, stiff insoles shifted the ankle-foot into a higher gear setting more suitable for fast walking, which may have contributed to the metabolic reduction at fast speeds. A similar speed-dependent effect of stiff insoles on metabolic cost has been found in a studying involving running^25^.

We investigated some alternative explanations as to how our insoles achieved a speed-dependent effect on metabolic cost. When looking at more proximal leg joints, hip and knee positive work both increased with the addition of stiff insoles, with hip work being equal to or greater than the amount of ankle work observed (Supplementary Fig. 5). However, no joints observed had a significant speed*stiffness interaction effect on positive work that mirrored the speed-dependent effect insoles had on metabolic cost. It is possible that muscle activations of knee and hip muscles could display the speed-stiffness interactions, however, we did not record electromyography data from these muscles. We also observed that insoles increased ground contact time on average by 2.58% (Supplementary Fig. 13) and this should have beneficial effects on rate of force production^26^ of the lower extremity muscles.

There are several other potential mechanisms that could affect metabolic cost with insoles. First, it is possible that the metabolic reduction at fast walking speeds was more related to elastic energy return from the carbon fiber insoles. We did see a slight increase in positive foot work with insoles (Supplementary Fig. 6), however positive work from the foot was greatest in the medium stiffness condition, which did not correspond to the greatest metabolic reduction. Thus, it is likely that the metabolic benefit afforded by the added foot stiffness conditions were more related to the increase in lever arm (or gear ratio) more so than the enhanced energy return from the foot. Second, the stiff insoles could have influenced the amount of energy stored and returned by the Achilles tendon. We did find that the ankle dorsiflexion range of motion increased due to stiff insoles, with a small change in Soleus fascicle length. This could indicate that the Achilles tendon lengthened and stored more energy when walking with stiff insoles. However, we did not directly measure tendon excursion in this study, making definitive conclusions about tendon’s work contributions difficult^27^. Lastly, the insoles could also alter demands on the muscles crossing the subtalar joint that contribute to frontal plane stability^28^ or on foot intrinsic muscles. Future studies analyzing activations from these muscles, and/or detailed foot musculoskeletal models may be needed to address these potential mechanisms.

A 7.1% reduction in energy cost of fast walking in our study is comparable to other devices that have also achieved energy cost reduction. For example, an ankle exoskeleton developed by Collins *et al*., composed of springs and clutches, reduced energy cost of walking by an average of 7.2% at normal walking speeds^29^. Similar metabolic cost reductions have also been observed in running, including a spring-loaded exoskeletons assisting at the hip (~8%)^30^ and an ‘exotendon’ that connected the two feet with a spring (~6.4%)^31^. Additionally, our 7.1% reduction in fast walking is much greater than previous results by Roy and Stefanyshyn, achieving only ~1% reduction with similar passive insoles during running^21^. Our metabolic reductions are also greater than those achieved by Hoogkamer *et al*.^18^ (~4%) and Oh & Park^32^ (~1.1%), who achieved cost reductions by altering longitudinal bending foot stiffness. While the metabolic benefit afforded by our passive insoles are less than various powered exoskeleton technology^33–37^, our findings confirm the mounting evidence that passive devices can offer metabolic reductions given that the device can interact effectively with the underlying muscles. Interestingly, some exoskeletons are thought to reduce metabolic cost by partially offloading the force demands of the muscles^29,38^. However, it is likely that this mechanism for metabolic reduction at fast walking speeds with stiff insoles is different. Rather than *decreasing* muscle force, stiff insoles can *increase* force output while improving the economy of muscles producing force.

Our stiffest insoles ranged from 61 to 92 grams from the smallest to largest sizes. In our present study, we added mass to control for the well-documented effects of distal mass on metabolic economy^39^, but it should be noted that comparing insole results to standard shoes without added mass would have more application to daily life. Using regression equations of added metabolic cost from mass placed on the foot^40^, if our insoles were compared versus shoes with no added mass, we could expect metabolic reduction to still be between 6.49% and 6.18% at 2.0 m/s walking, depending on the foot size of subject. This is still an amount comparable to other exoskeleton and assistive device studies of the present time^29,30^.

We have some limitations in our study. The current experiment separated collection days into two separate visits to the lab; however, these visits were counterbalanced across subjects to control for learning effects or familiarization between sessions. It is unclear whether additional days of familiarization could affect metabolic energy cost of walking with insoles. Our estimates of Soleus fascicle forces are based on assumptions about forces being proportional to the relative cross-sectional area among the plantarflexor muscles, and that co-activation from antagonistic muscles is minimal. In addition, our plantarflexor moment arm estimates (which are required for the force estimates) do not account for the changes that may occur as a function of loading during stance^41^. While such assumptions affect the overall magnitude of force estimates, our main finding that stiff insoles shifted the force-velocity operating region of the Soleus muscle is also corroborated from our data showing increased ankle plantarflexion moment with greater insole stiffness (Supplementary Figs. 2 and 3) without greater activation from the plantarflexor muscles including the Soleus, and medial and lateral Gastrocnemius (Supplementary Figs. 10 and 11). Lastly, there are limitations with using ultrasound imaging to quantify muscle fascicle behavior, including that we are taking a two-dimensional image of a three-dimensional muscle. It is also likely that the accuracy and reliability of the images are operator dependent. These limitations were mitigated by utilizing a single session within-subjects design, and following best practices as outlined by Farris *et al*.^42^ by not changing probe placement across conditions and tracking all ultrasound data with the same investigator.

Overall, we found that stiff insoles augmented functions of the major ankle plantarflexor muscle (Soleus) during fast walking, a locomotion paradigm in which muscles appear to function sub-optimally due to task constraints. This shifting of force-velocity operation of ankle muscles could be beneficial in other situations or in other subject populations where the plantarflexors are hindered in performance. For example, load-carriage or incline walking tasks require increased force output from the plantarflexors^43^, which may be assisted by stiff insoles. Future studies using stiff insoles could also examine the preferred walk-to-run transition speed of subjects, as increased economy of force production at fast speeds may expand the available speeds in which the human body can comfortably walk. Lastly, it may be possible that individuals who have deficient ankle push-off mechanics, such as elderly adults^44,45^, could benefit from insoles to augment force capacity.

## Conclusions

This study utilized a passive, lightweight device (i.e., carbon-fiber shoe insoles) to alter the gearing of the foot and ankle. In particular, stiff insoles decreased ankle joint plantarflexion and increased dorsiflexion range of motion, decreased Soleus fascicle shortening velocity, and increased force output, without greater muscle activation. The stiff insoles had a speed-dependent influence on metabolic cost, where at normal walking speeds, we observed a 9.6% increase in metabolic cost, and at fast walking speeds we observed a 7.1% cost reduction. By utilizing the gearing mechanisms of the foot and ankle, and targeting tasks that induce sub-optimal function of the plantarflexor muscles, we were able to break the normal energy barrier in fast walking. It is likely that optimal foot stiffness is locomotor task dependent, and foot-ankle musculature as well as external assistive devices all play a role in shifting gears for different tasks. The simple, passive insoles we used in this study seem to add an extra gear unavailable to the human foot-ankle system, a gear which is beneficial at fast speeds.

## Materials and Methods

### Insoles and shoes

Insoles used to add foot stiffness consisted of either 1.6 mm or 3.2 mm carbon fiber plates, cut out in the shape of 9 different shoe inserts (Women’s Euro sizes 37, 38, and 39; Men’s Euro sizes 42, 43, 44, 45, 46) previously used in Takahashi *et al*.’s study^13^. Insoles were designed to fit underneath the normal insole of our shoe and increase stiffness without coming into direct contact with the skin. Three-point bending tests performed similar to previous research^13,19,21^ quantified longitudinal bending stiffness of control shoes (Reebok RealFlex Train) in three stiffness conditions: shoes alone (*low*), shoe+1.6 mm insole (*medium*), and shoe+3.2 mm insole (*high*). During low and medium stiffness conditions, lead tape was added to the shoes to equalize mass to the high stiffness condition to account for the confounding factor of foot/shoe mass on metabolic cost^39,40^.

### Participants

15 healthy young adults (N = 13 males, 2 females, age 23 ± 2.1 yrs, height 176 ± 7.3 cm, mass 76.4 ± 12.4 kg) participated in this study, conducted at the University of Nebraska at Omaha under the approval of the Institutional Review Board of the University of Nebraska Medical Center. Informed consent was obtained from all subjects involved in the study, and methods were carried out in accordance with the study’s IRB-approved protocol and followed guidelines of the Declaration of Helsinki. Subjects completed two visits to the research lab, referred to as a *mechanics visit*, and a *metabolics visit*. During each session, participants walked in three different walking speeds (1.25, 1.75, and 2.0 m/s) and three different stiffness conditions (low, medium, and high) for a total of 9 conditions (Fig. 2). Sessions were separated to ensure patient comfort while walking with the ultrasound collection equipment. The order of the two visits was randomized to each subject, to counterbalance for possible learning effects across visits.

### Experimental protocol

On one visit, subjects walked for a brief time at each condition, during which three sets of ten steps of data were collected for analysis. Subjects were given approximately 30 seconds to familiarize to each walking speed and stiffness conditions before data were collected. Three-dimensional limb kinematic (i.e., motion) data were captured using an eight-camera motion capture system (VICON, Oxford, UK), as subjects walked on an instrumented treadmill (Bertec, Columbus, OH, USA) to capture limb kinetics (i.e., forces). A six-degree-of-freedom (6DOF) marker set was used^46^ to track motion of the lower extremities. Electromyography (EMG) sensors (Delsys, MA, USA) were placed on the Tibialis Anterior, Medial Gastrocnemius, Lateral Gastrocnemius, and Soleus of the left leg to record muscle activity (N = 14, one subject EMG removed due to technical difficulties). Lastly, the subjects had a 60 mm linear ultrasound probe (Telemed LV7.5/60/128Z-2, Lithuania) secured over a region of the Lateral Gastrocnemius on the right leg, allowing an *in vivo* view of the deeper Soleus muscle fiber contractions. During the other visit, subjects walked for 6 minutes at each of the 9 conditions (3 speeds × 3 stiffnesses), while breath-by-breath gas exchange measurements were recorded for indirect calorimetry calculations of metabolic cost (Parvo Medics, Sandy, UT, USA). Metabolics data were collected early in the morning, before subjects ate breakfast, to control for effects of dietary intake on metabolic data.

### Analysis of gear ratio

Gear ratio – the ratio between ground reaction force moment arm and biological plantarflexor moment arm – was calculated over the duration of the stance phase. Subject-specific plantarflexor moment arm was determined as a function of dorsiflexion angle using a technique used in previous research^13,47^. We took a photograph of each subject’s ankle in a neutral position on a lined reference block, and then digitized the image to measure neutral Achilles tendon moment arm. After that, a general regression model derived from previous imaging literature^48^ calculated subject-specific moment arm during stance. Gear ratio was only averaged between 5–95% of stance, as moment arm calculations are error prone at low magnitudes of ground reaction forces.

### Analysis of ankle range of motion

From the ankle joint angle data, we quantified dorsiflexion range-of-motion (ROM) as the difference between peak dorsiflexion angle during stance and minimum of ankle angle during the first half of stance. Similarly, plantarflexion ROM was quantified by as the difference between peak dorsiflexion angle and the peak plantarflexion angle.

### Analysis of fascicle velocity

A flat, linear ultrasound probe (Telemed, Lithuania) was secured to the subject’s lower leg, superficial to the ankle plantarflexor muscles, then rotated and translated until a clear image of a Soleus fascicle was visible on screen, corresponding to when fascicle and probe were within the same plane. Soleus fascicle contractions were captured at approximately 78 Hz. This method for recording muscle fascicle behavior has been previously proved reliable and accurate^9,49–51^. We used a semi-automated tracking algorithm^42^ to quantify length of fascicles over time, which can be differentiated to achieve Soleus fascicle velocity. The tracking software has been used previously for measuring length and pennation angle of the ankle plantarflexors^42,52^ across different walking speeds^9^ and in added foot stiffness conditions^13^. Shortening velocity of Soleus fascicles was analyzed both as an average over stance phase as well as the instantaneous velocity at the time of maximum Soleus force generation.

### Analysis of soleus fascicle force

We estimated Soleus muscle force by combining inverse dynamics approaches, modeling-based divisions of muscle forces, and subject-specific ultrasound imaging^13,38^. Inverse dynamics calculated net ankle moment was divided by ankle plantarflexion moment arm (acquisition method discussed above) to estimate overall plantarflexor force. This force was scaled by the cross-sectional area of the Soleus relative to other plantarflexors (0.54), derived by medical imaging studies^53^. Lastly, this Soleus force was scaled by the cosine of time-varying, subject-specific pennation angle, measured from the ultrasound data (described previously).

### Analysis of metabolic cost

Whole-body Metabolic power was calculated using standard equations derived by Brockway^54^. For all metabolic trials, only the last two of six minutes of calorimetry data were averaged for calculations, to allow time for whole-body metabolic rate to stabilize. Firstly, a six minute quiet standing trial was conducted to approximate metabolic cost of standing. For all following trials, we calculated net normalized metabolic power (divided by subject body mass) by subtracting metabolic cost of quiet standing. Metabolic cost of transport was then calculated by dividing net metabolic power by walking speed, giving a measure of whole-body metabolic energy expenditure per unit distance traveled.

### Additional analyses

Kinematic and kinetic data were sampled at 250 and 1000 Hz, respectively, and filtered with 6 and 25 Hz 2^nd^ order low-pass Butterworth filters, as previously used with data of this nature^13^. Kinematic and kinetic data were combined to calculate six-degree-of-freedom joint powers at the ankle, knee and hip; this approach has been shown to more accurately estimate energy changes of the whole body^55^. Foot deformation power was estimated using a single segment foot model consistent with a prior study^13^. Electromyography (EMG) data were high-pass filtered at 20 Hz, rectified, and then low-pass filtered at 10 Hz with second-order Butterworth filters to achieve a linear envelope. EMG data were then time-integrated from heel strike to toe off to quantify amount of muscle activation over stance.

### Statistical analysis

A two-factor, two-tailed repeated measures ANOVA was performed to test for main and interaction effects between walking speed and foot stiffness on our kinetic, kinematic and metabolic outcome variables: gear ratio (stance-averaged and peak), ankle range-of-motion, fascicle force (stance-averaged and peak), fascicle length (stance-averaged and at the time of peak force), fascicle velocity (stance-averaged and at the time of peak force), muscle activation (integrated during stance, and peak), and metabolic cost of transport. Significant (p < 0.05) main effects confirmed if walking speed or foot stiffness had an effect on outcomes. When interaction effects were present, we used post-hoc pairwise comparisons between each stiffness condition within our three speeds using Fisher’s Least Significant Difference adjustment. Significant pairwise comparisons determined which conditions differed from each other in our outcome variables.

## Supplementary information

Supplementary Information.

## Data Availability

Data from the current study are available from the corresponding author on reasonable request.
