# Supplementary material for: Gearing Up the Human Ankle-Foot System to Reduce Energy Cost of Fast Walking
Source: Sci Rep. 2020 May 29;10:8793. doi: 10.1038/s41598-020-65626-5 (PMC7260196; doi:10.1038/s41598-020-65626-5)
Supplement: Supplementary file 1 — Supplementary Information. [file 41598_2020_65626_MOESM1_ESM.pdf]

Supplementary Information

Gearing Up the Human Ankle-Foot System to Reduce Energy Cost of Fast Walking

Samuel F. Ray

Kota Z. Takahashi

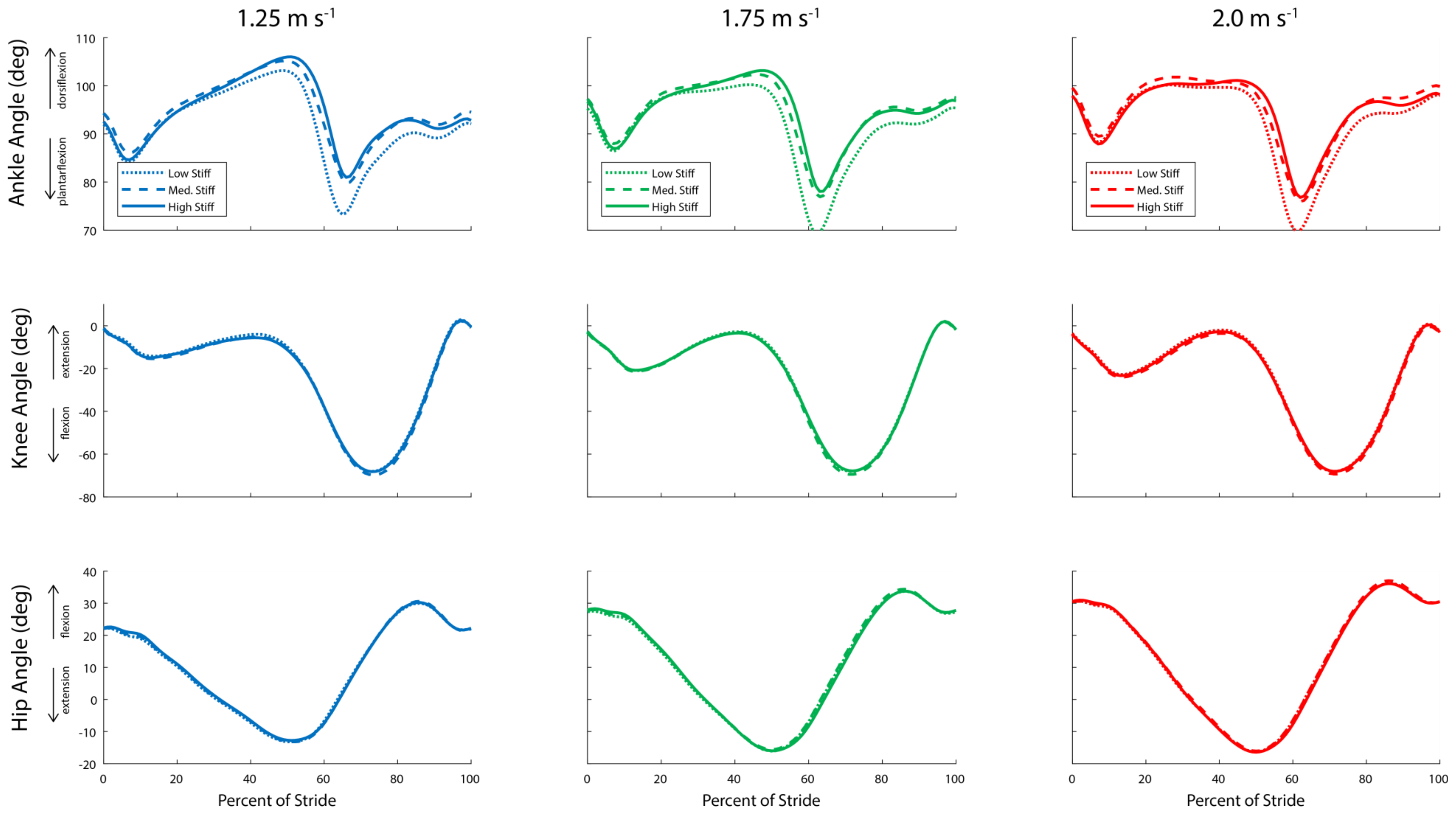

Supplementary Figure 1: Stride-normalized joint angles.

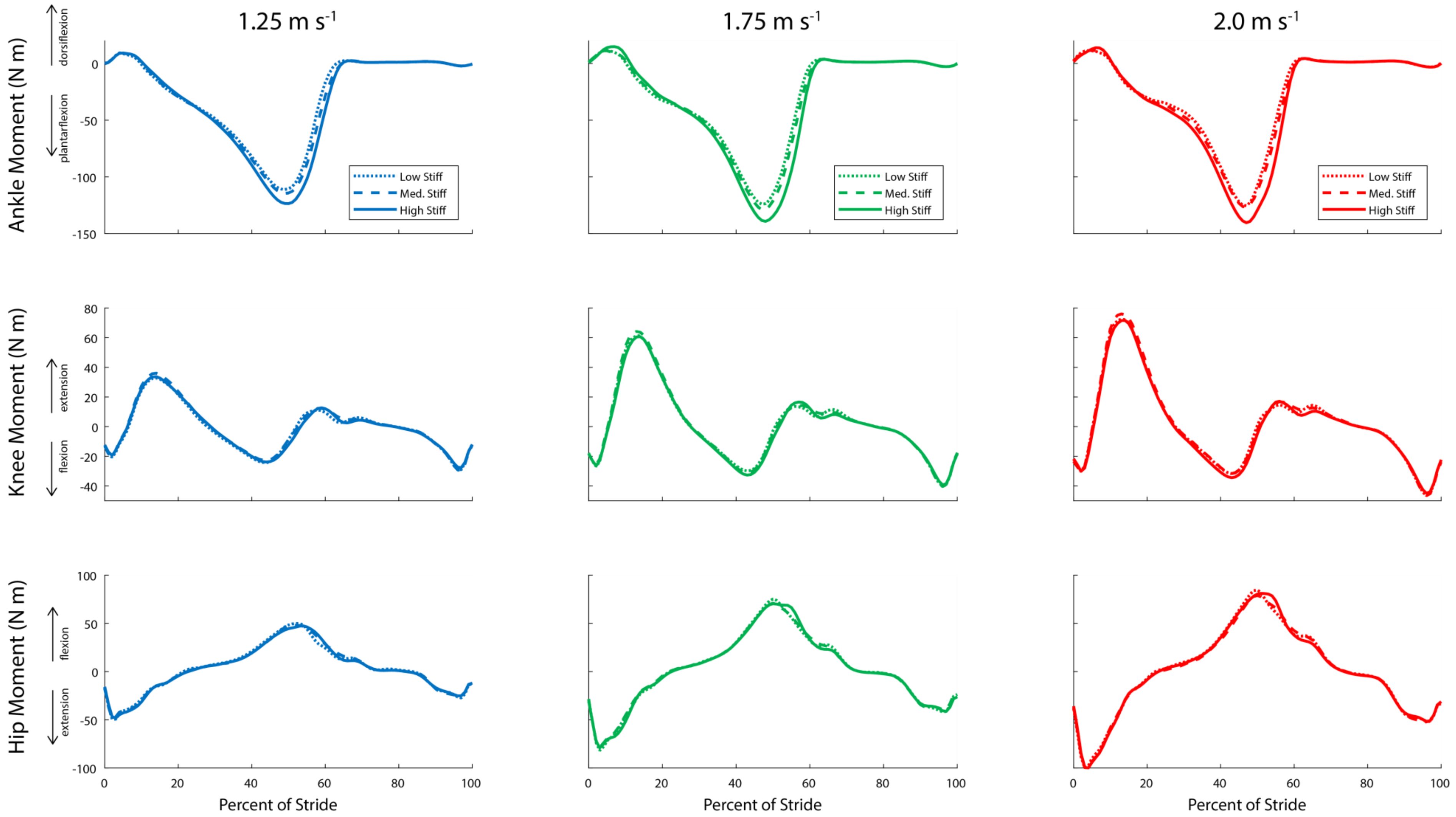

Supplementary Figure 2: Stride-normalized joint moments.

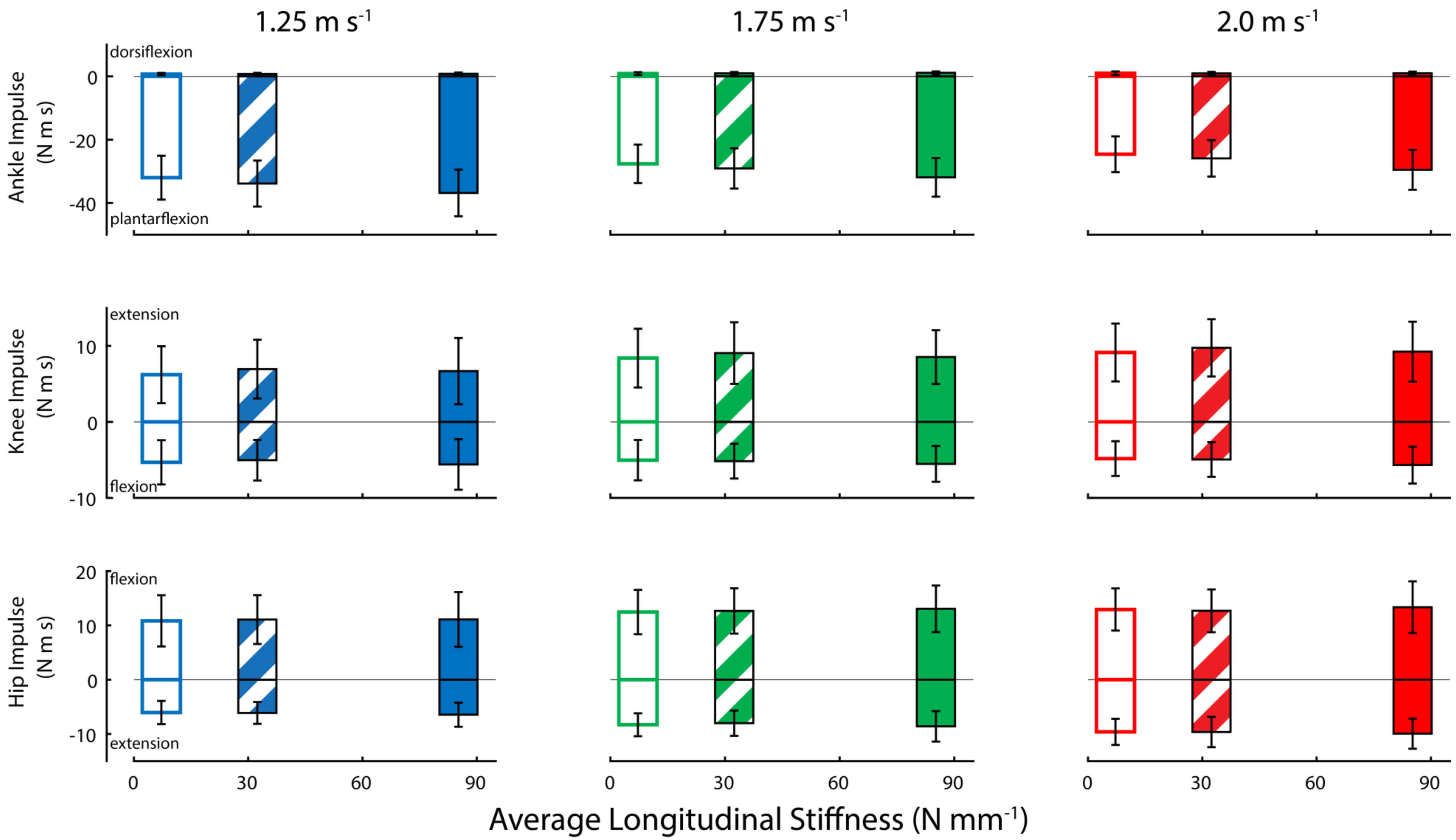

Supplementary Figure 3: Stance-integrated joint moments. Significant main effects of walking speed were observed in: ankle dorsiflexion impulse (df=2, F=7.93, p=0.002), ankle plantarflexion impulse (df=2, F=90.80, p<0.001), knee extension impulse (df=2, F=19.41, p<0.001), hip flexion impulse (df=2, F=18.19, p<0.001), and hip extension impulse (df=2, F=85.61, p<0.001). Significant main effects of foot stiffness were observed in: ankle dorsiflexion impulse (df=2, F=3.78, p=0.035), ankle plantarflexion impulse (df=2, F=112.13, p<0.001), knee extension impulse (df=2, F=7.09, p=0.003) and knee flexion impulse (df=2, F=13.08, p<0.001). No interaction effects were observed at any joint. Error bars signify s.d.

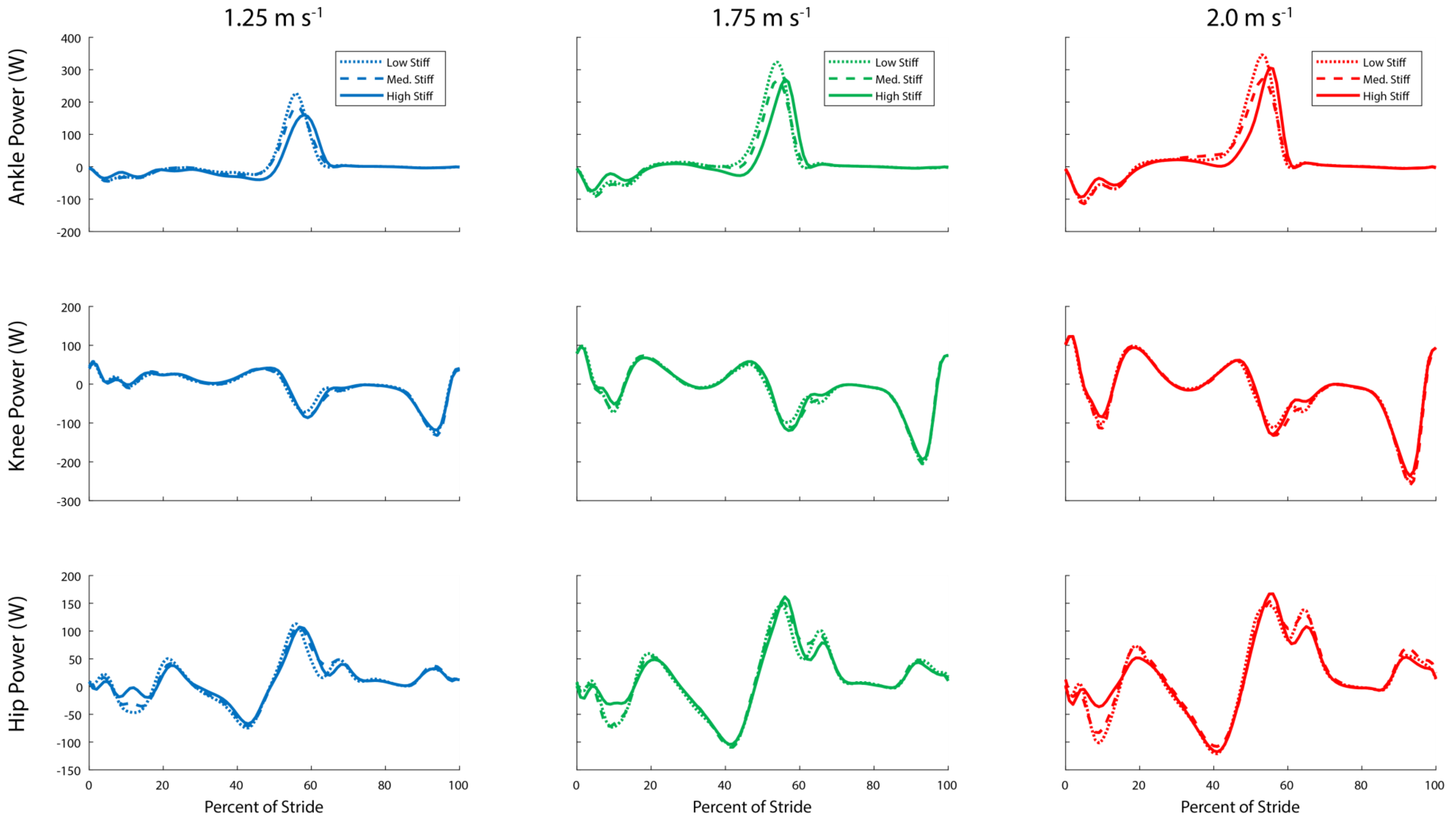

Supplementary Figure 4: Stride-normalized six-degree-of-freedom joint powers.

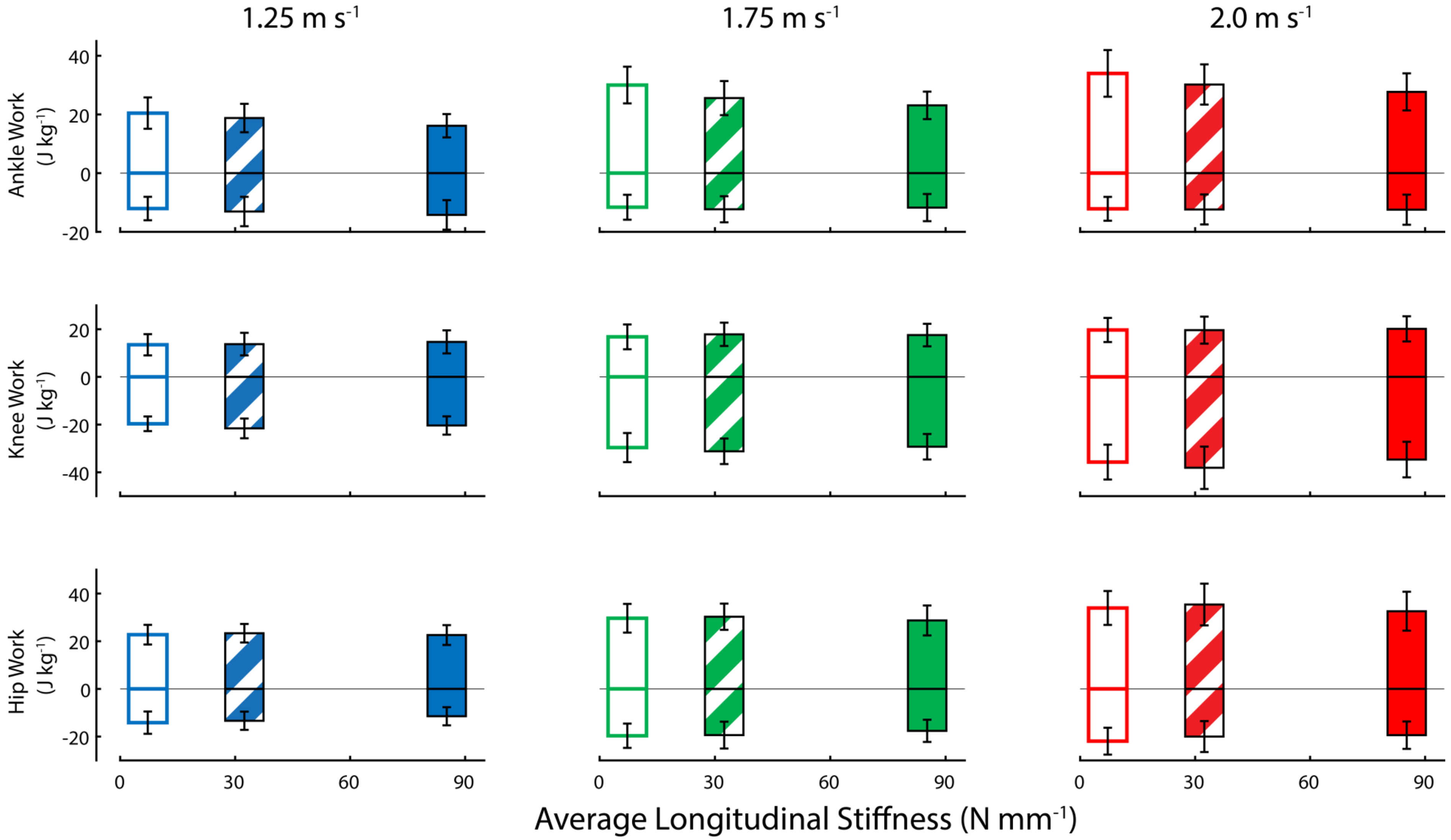

Supplementary Figure 5: Stride-integrated six-degree-of-freedom joint works. Significant main effects of walking speed were observed in: positive (df=2, F=127.02, p<0.001) and negative (df=2, F=3.53, p=0.043) ankle work, positive (df=2, F=67.52, p<0.001) and negative (df=2, F=153.4, p<0.001) knee work, and positive (df=2, F=46.28, p<0.001) and negative (df=2, F=63.98, p<0.001) hip work. Main effects of stiffness were observed in: positive ankle work (df=2, F=42.18, p<0.001), positive (df=2, F=3.65, p=0.039) and negative (df=2, F=26.09, p<0.001) knee work, and positive (df=2, F=5.12, p=0.013) and negative (df=2, F=20.87, p<0.001) hip work. There were significant interaction speed*stiffness effects for negative work at the ankle (df=4, F=3.20, p=0.020), knee (df=4, F=2.69, p=0.040), and hip (df=4, F=2.64, p=0.043) joints. Error bars signify s.d.

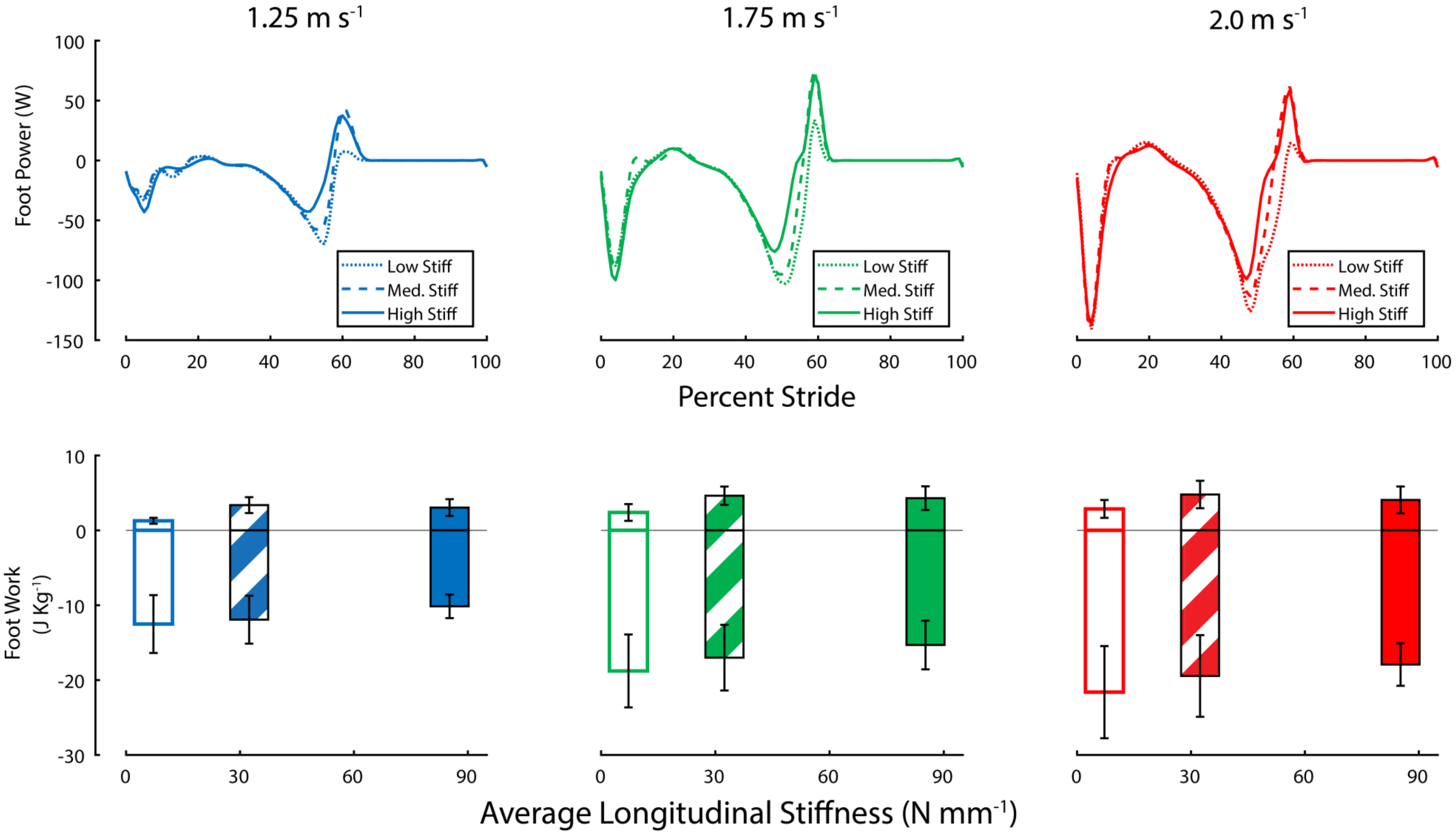

Supplementary Figure 6: Foot power and work. Positive foot work exhibited a main effect of walking speed (df=2, F=21.75, p<0.001) and foot stiffness (df=2, F=37.24, p<0.001). Negative foot work also exhibited main effects of walking speed (df=2, F=126.83, p<0.001) and foot stiffness (df=2, F=8.81, p=0.001). Stiff insoles showed reduced energy dissipation at the foot, as well as a slightly greater energy return. However, the medium stiffness condition exhibited greatest positive foot work output. We note that the foot power was estimated using a single segment foot model consistent with a prior study[1]. However, recent studies have shown that this approach may neglect positive power/work generated from other regions of the foot[2,3], such as the midtarsal joint (i.e., arch). Thus, our estimates of foot positive work reported here may underestimate the work contributions of all structures within the foot. Error bars signify s.d.

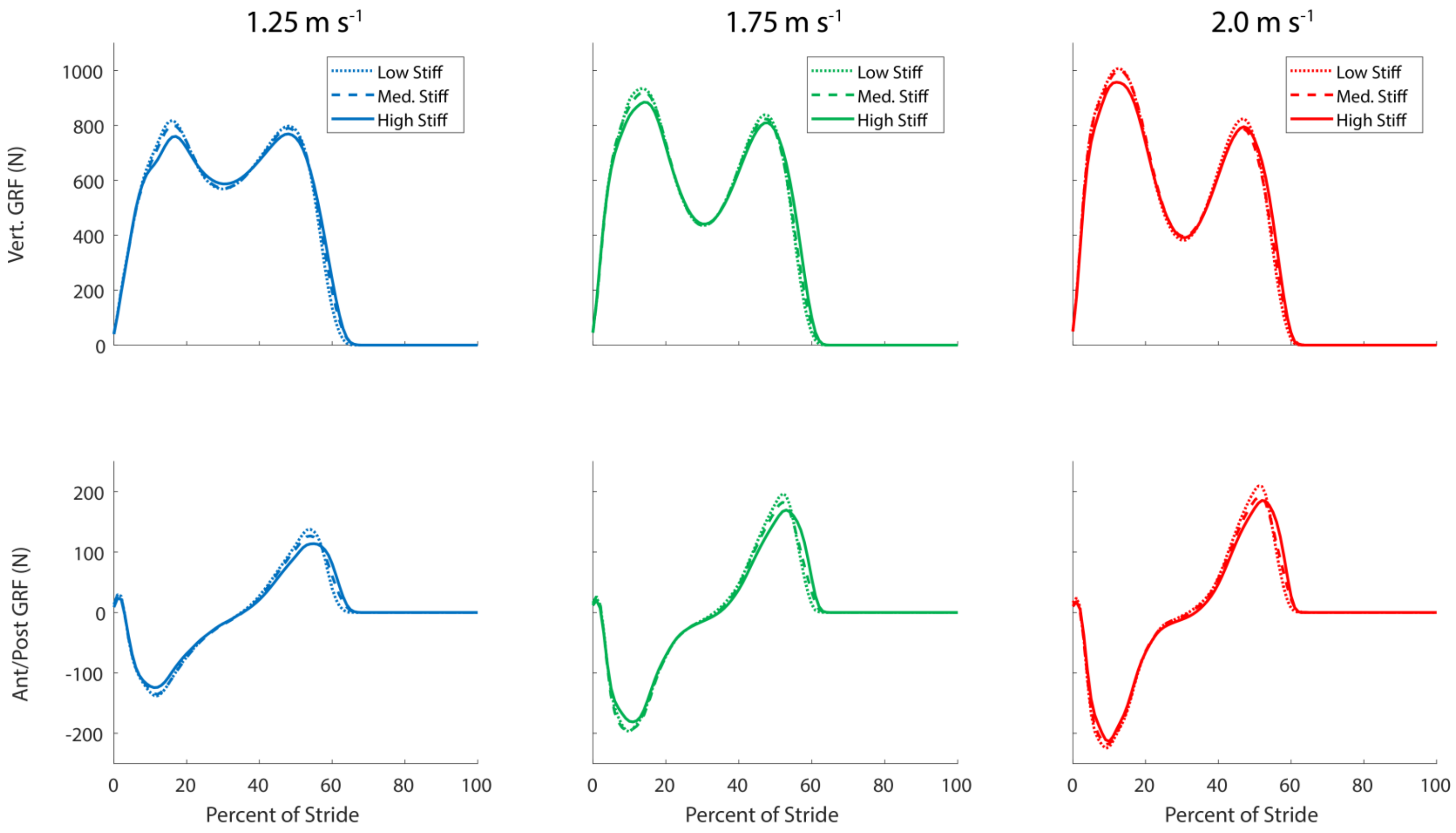

Supplementary Figure 7: Vertical and anterior ground reaction forces.

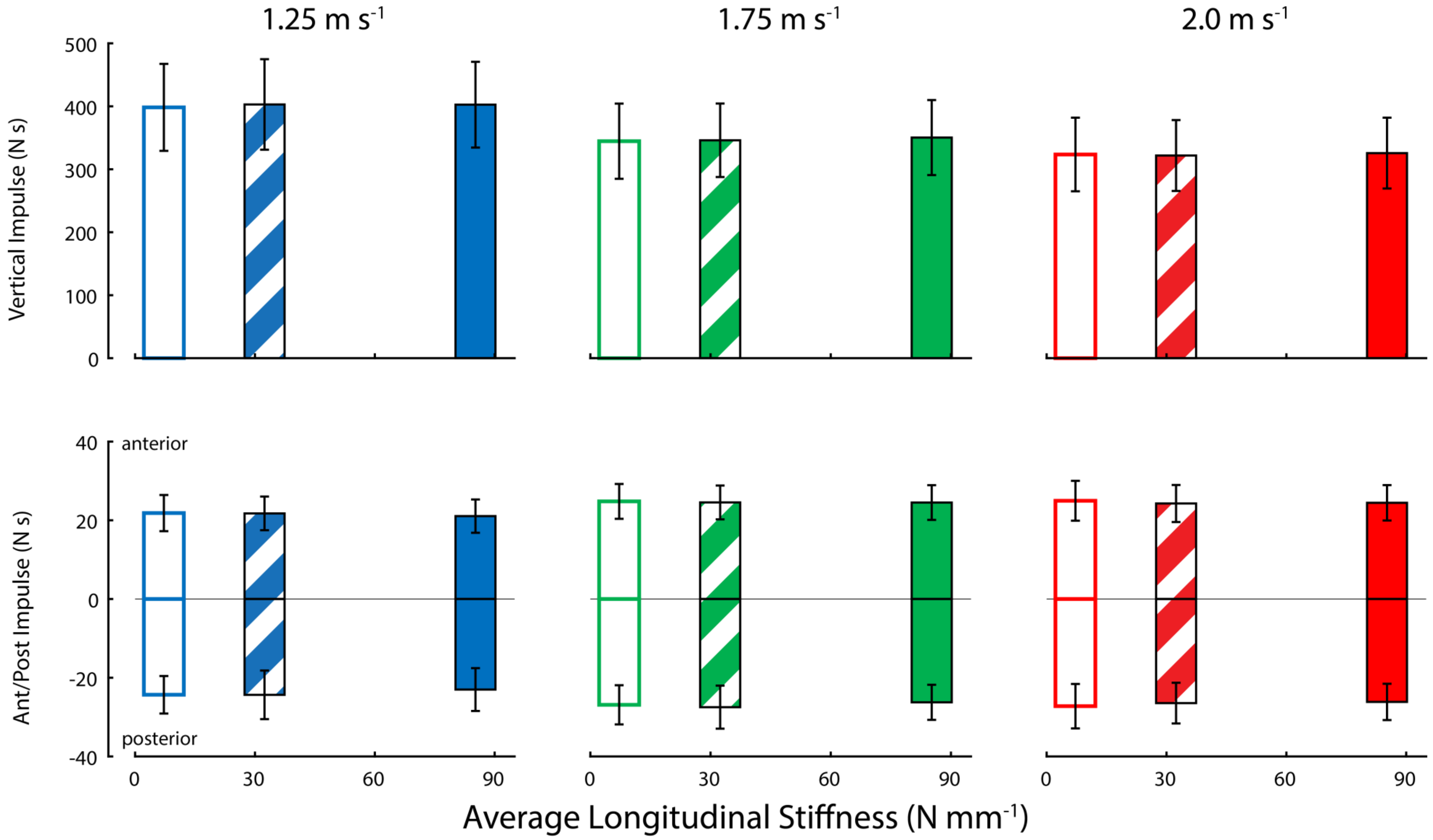

Supplementary Figure 8: Stance-integrated ground reaction force impulses. Significant main effects of walking speed were observed in vertical impulse (df=2, F=242.56, $p<0.001$), anterior impulse (df=2, F=22.11, $p<0.001$), and posterior impulse (df=2, F=10.52, $p<0.001$). No ground reaction force impulses exhibited significant main effects of foot stiffness or interaction effects. Error bars signify s.d.

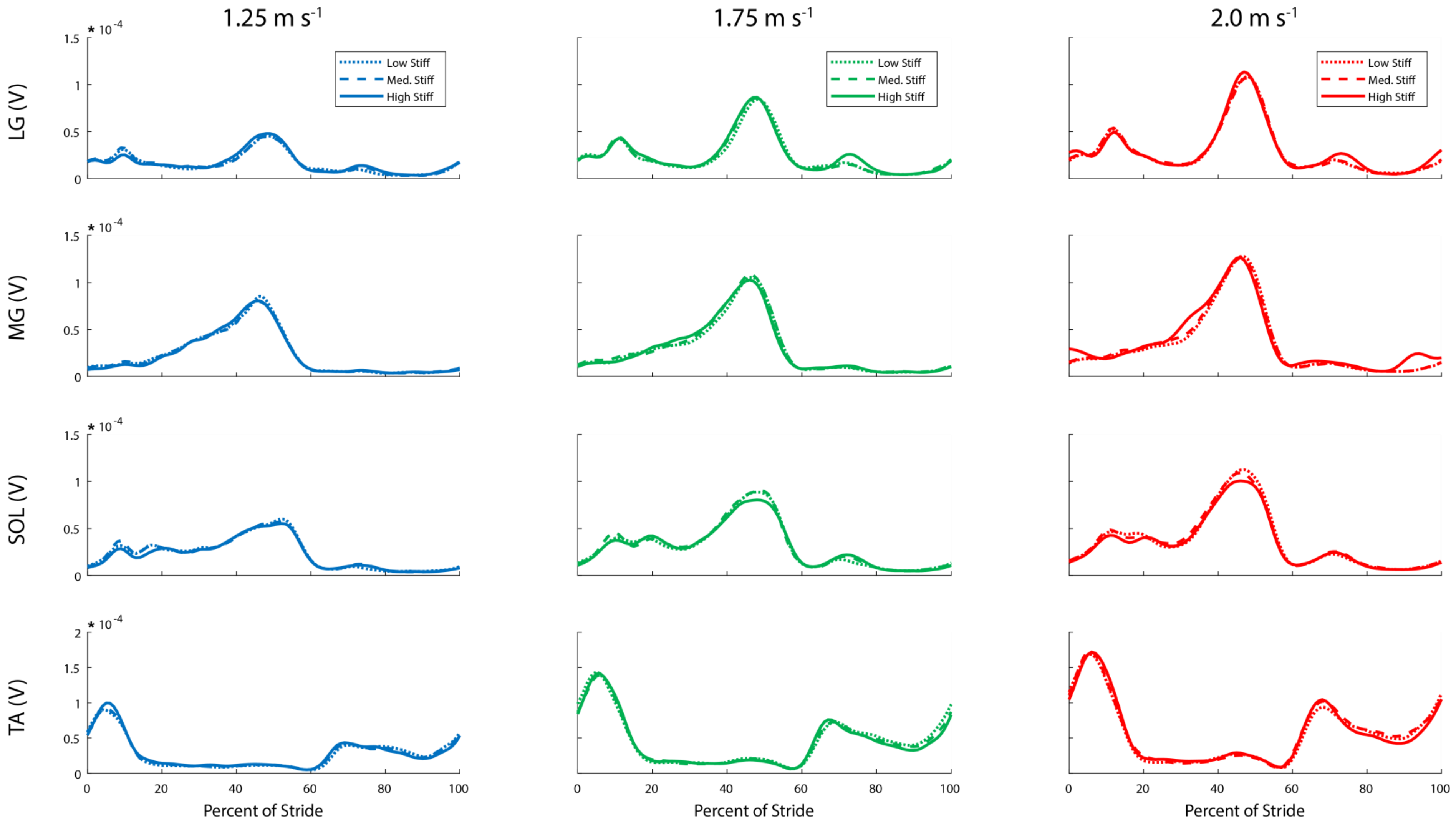

Supplementary Figure 9: Stride-normalized EMG traces of Lateral Gastrocnemius (LG), Medial Gastrocnemius (MG), Soleus (SOL), and Tibialis Anterior (TA) muscles (N = 14, Left leg)

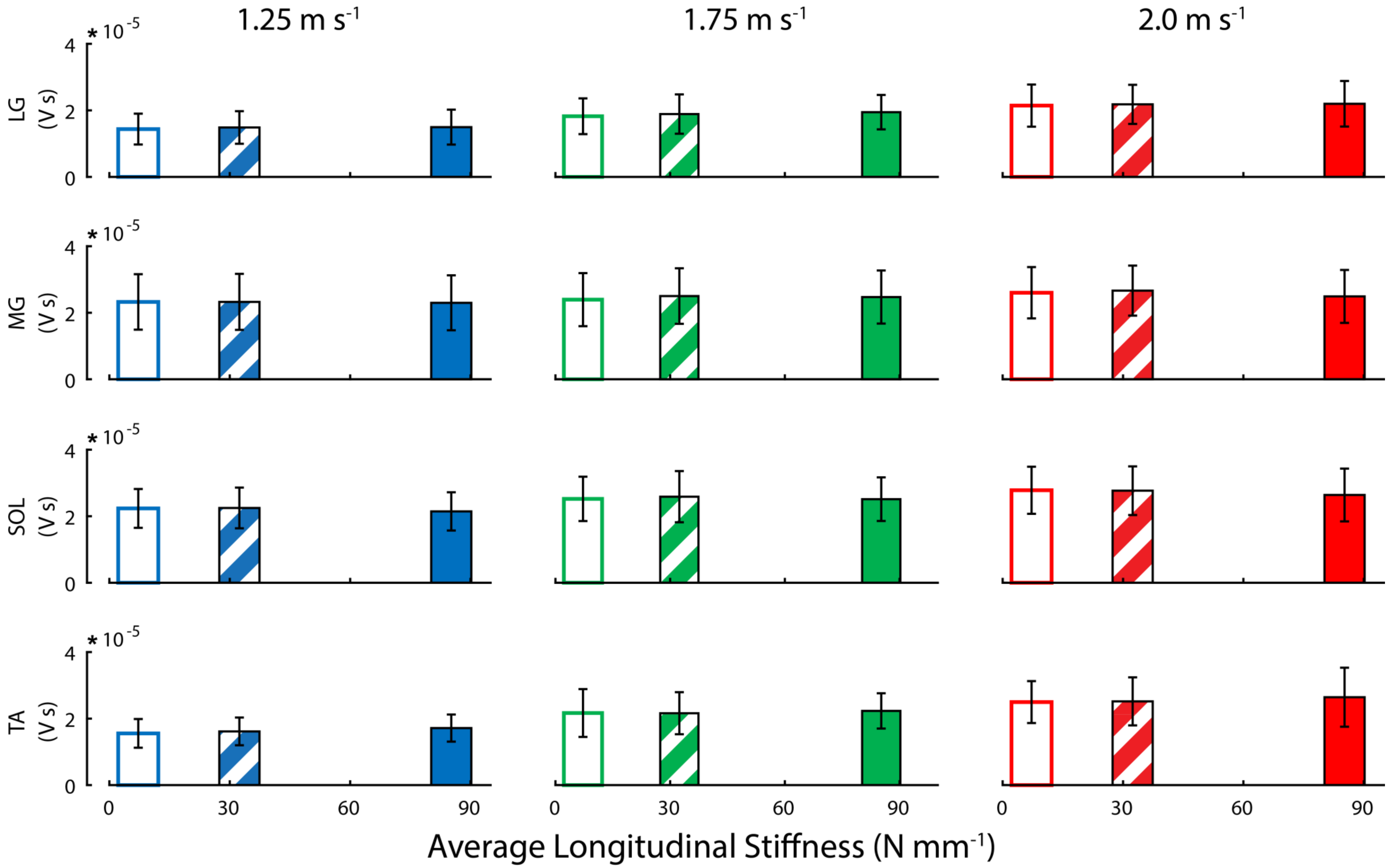

Supplementary Figure 10: Stance-integrated electromyography activations (N=14, left leg). Significant main effects of walking speed were observed in all four muscles - Lateral Gastrocnemius (LG) (df=2, F=48.48, p<0.001), Medial Gastrocnemius (MG) (df=2, F=8.72, p<0.001), Soleus (SOL) (df=2, F=30.05, p<0.001), and Tibialis Anterior (TA) (df=2, F=71.17, p<0.001). No foot stiffness or interaction effects were observed. Error bars signify s.d.

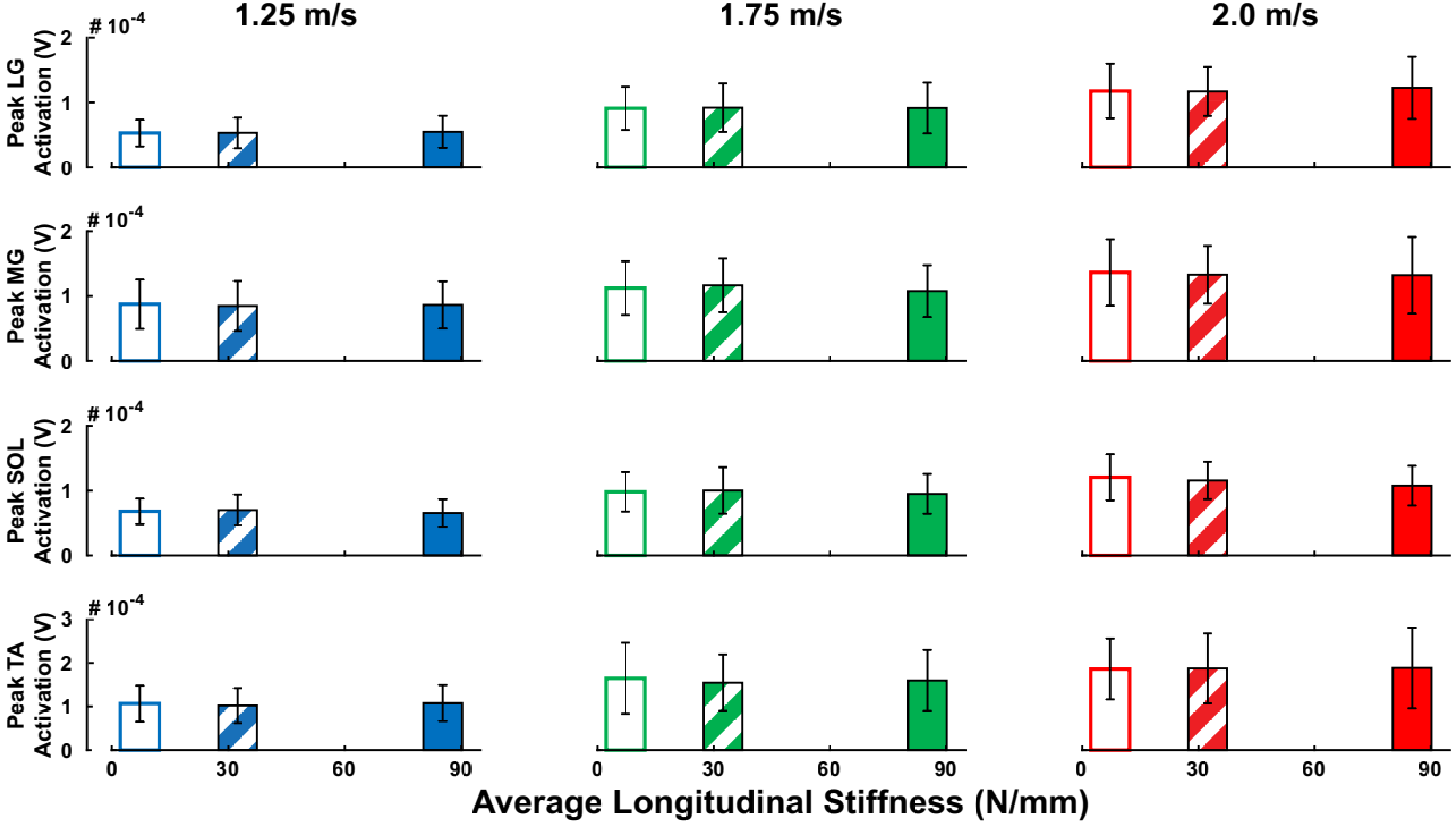

Supplementary Figure 11: Peak electromyography activations (N=14, left leg). Significant main effects of walking speed were observed in peak activation levels of all four muscles (all p<0.001). No foot stiffness or interaction effects were observed. Error bars signify s.d.

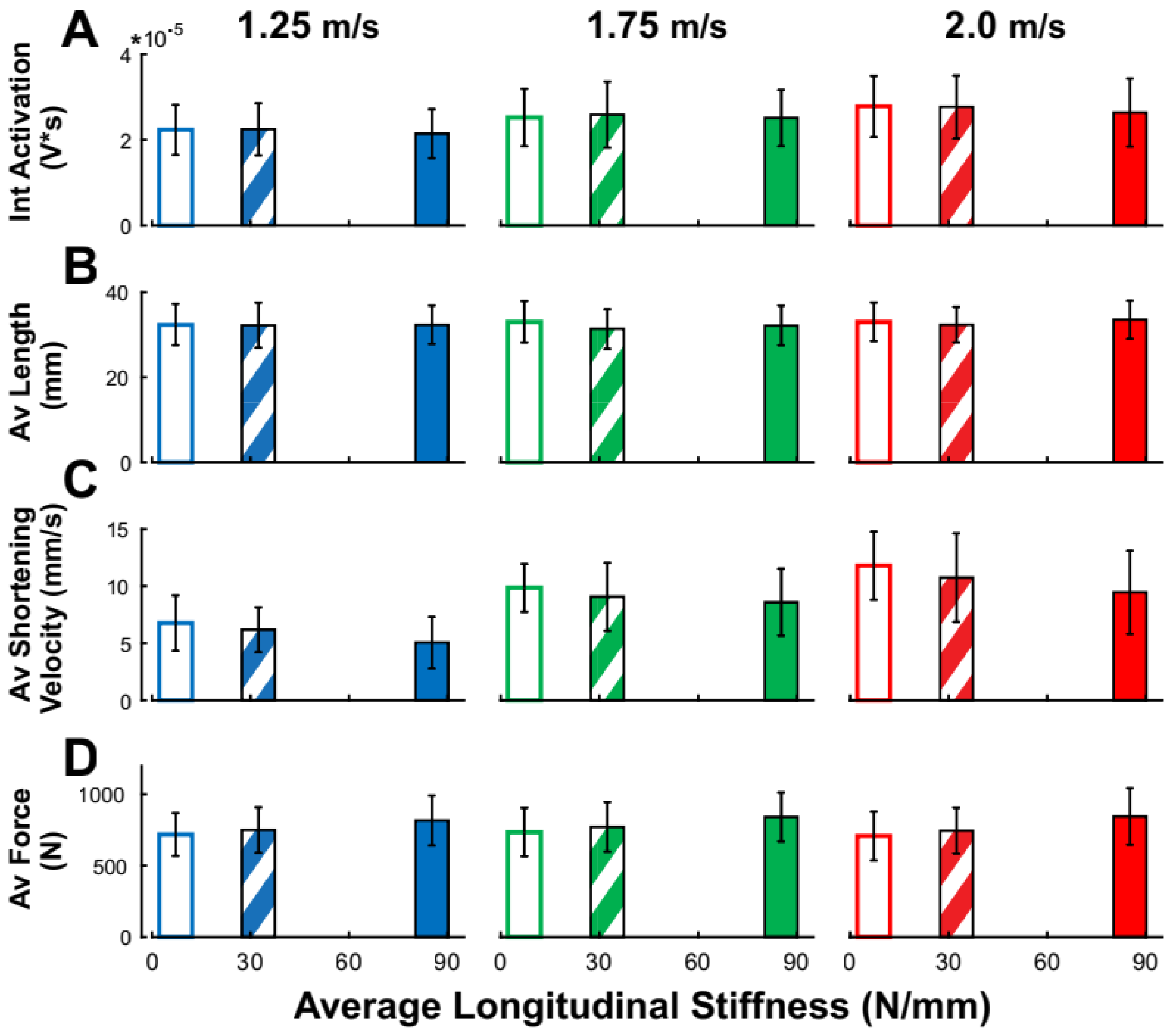

Supplementary Figure 12: Soleus muscle group mean data. A) Stance-integrated soleus activation increased with walking speed (p<0.001) but did not change with added stiffness (p=0.1448). B) Average fascicle length during stance did not change with added stiffness (p=0.067) or faster walk speeds (p=0.182). C) Stance-averaged fascicle shortening velocity increased with walking speed (p<0.001) and decreased with added foot stiffness (p=0.002). D) Average fascicle force during stance increased with stiffness (p<0.001) but did not change with speed (p=0.269). Error bars signify s.d. between subjects.

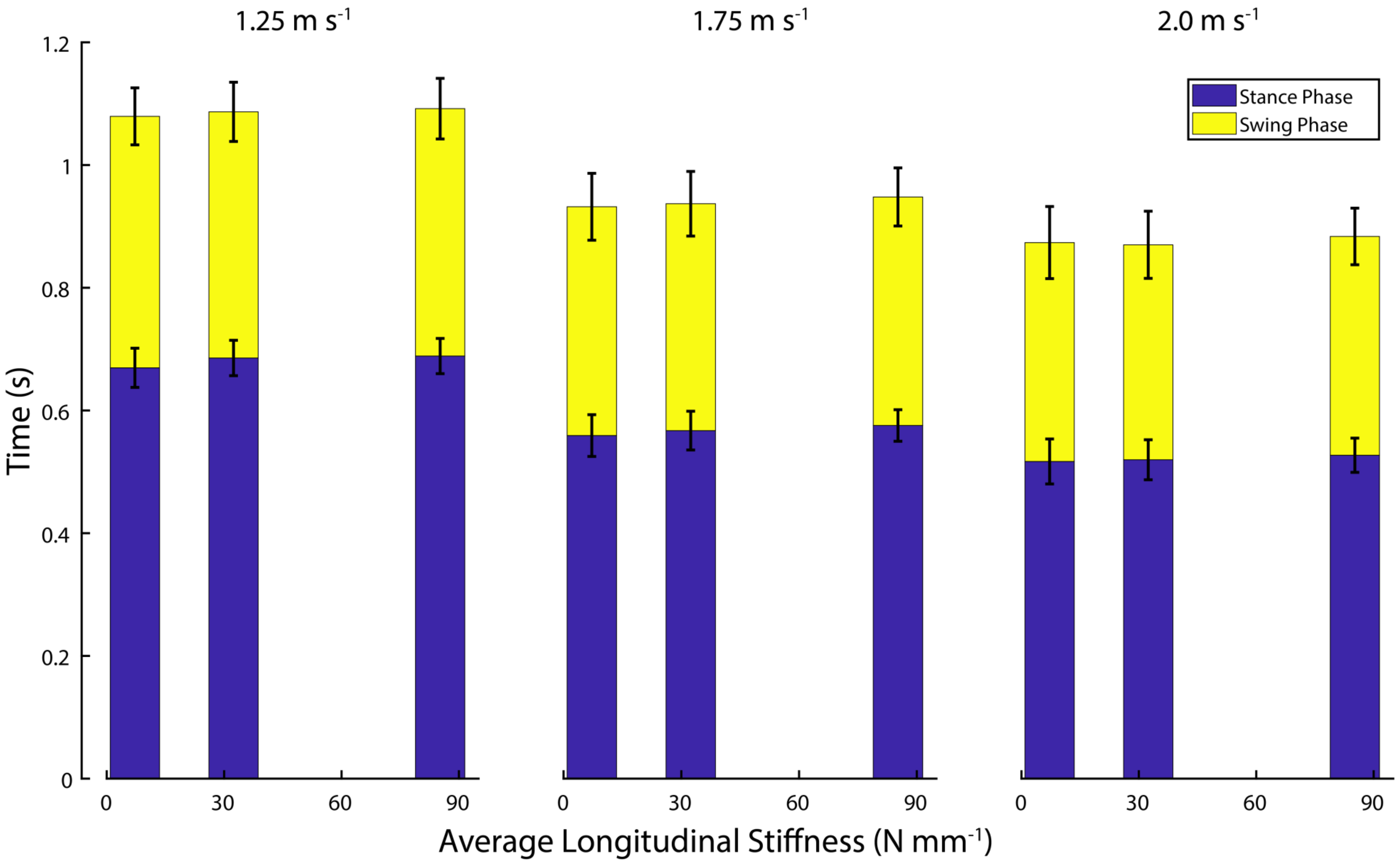

Supplementary Figure 13: Stance and swing time. Average stance time showed significant main effects of walking speed (df=2, F=1266.3, p<0.001) and foot stiffness (df=2, F=17.25, p<0.001), and stride time showed these same main effects as well (walking speed df=2, F=826.7, p<0.001, stiffness df=2, F=5.85, p=0.008). Stance time exhibited a speed*stiffness interaction effect as well (df=4, F=3.13, p=0.022). Error bars signify s.d.

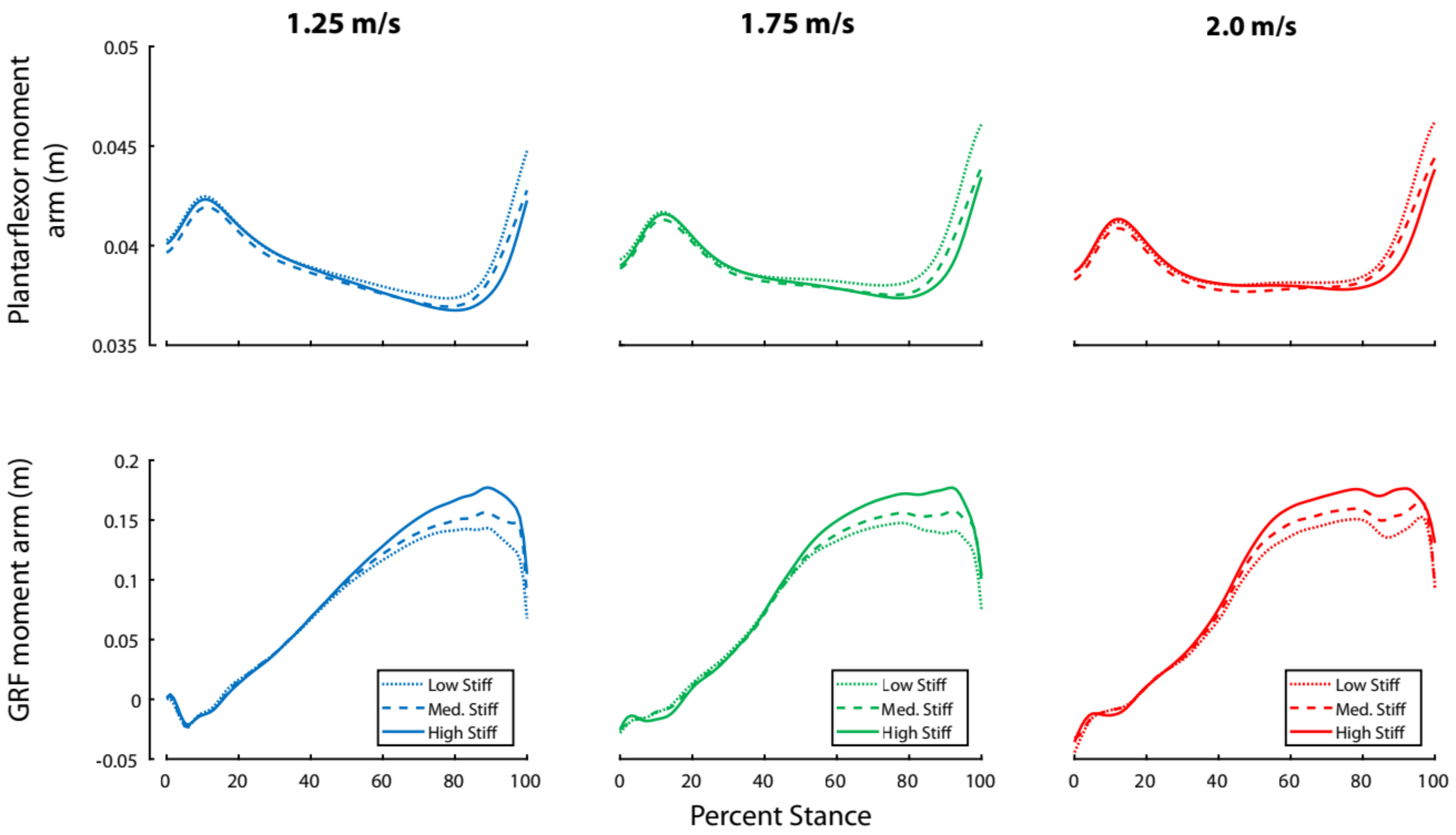

Supplementary Figure 14: Stride-normalized lever arms of plantarflexors (top) and Ground Reaction Force (bottom) about the ankle joint.

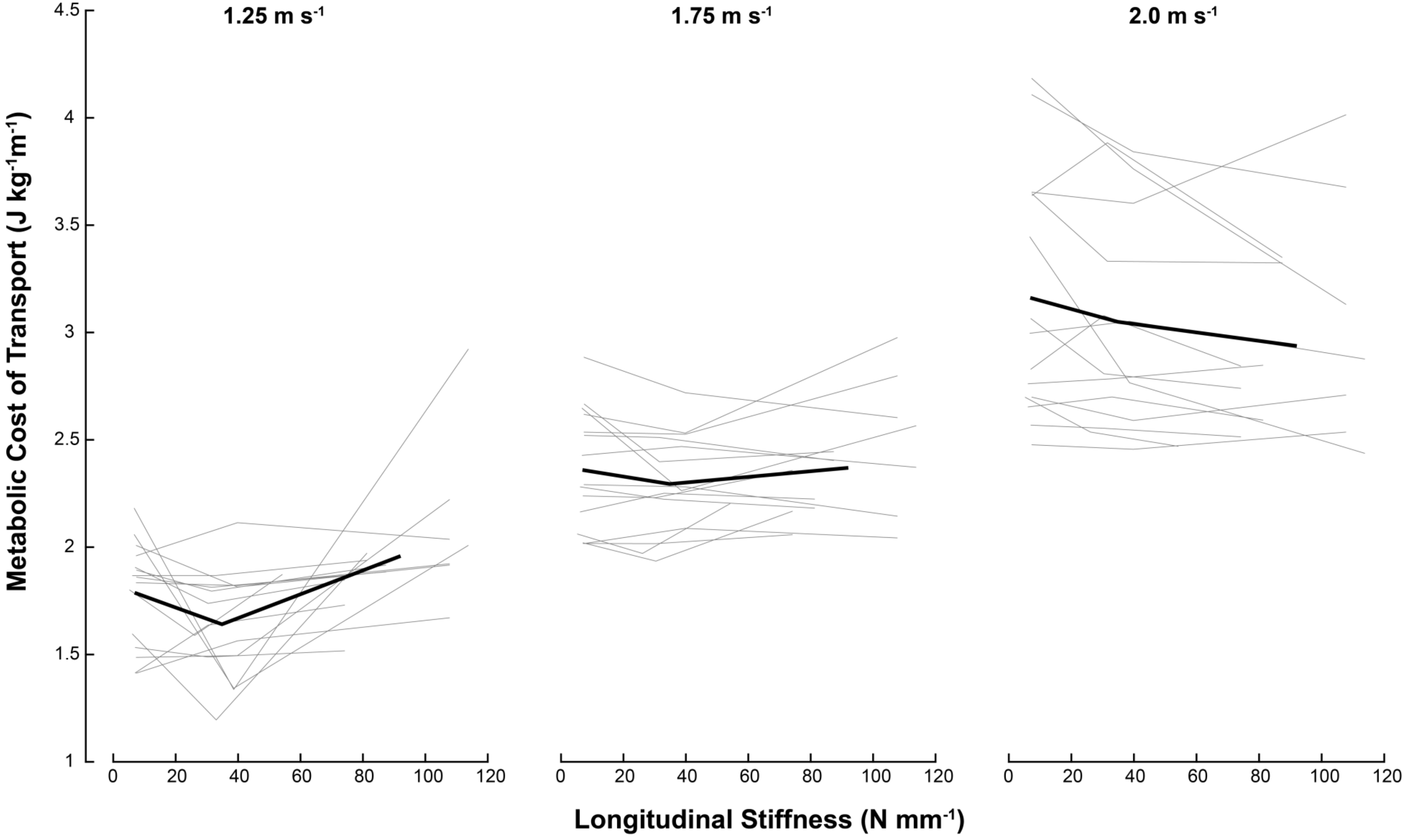

Supplementary Figure 15: Individual subject data for metabolic cost of transport and foot stiffness. Grey lines are each individual subject's three metabolic data plotted vs. their longitudinal foot stiffness for each trial. Longitudinal foot stiffness varied between subjects due to differing foot/shoe lengths. Black line represents group mean trend.

References

1. Takahashi, K. Z., Gross, M. T., van Werkhoven, H., Piazza, S. J. & Sawicki, G. S. Adding Stiffness to the Foot Modulates Soleus Force-Velocity Behaviour during Human Walking. *Sci. Rep.* **6**, (2016).

2. Takahashi, K. Z., Worster, K. & Bruening, D. A. Energy neutral: the human foot and ankle subsections combine to produce near zero net mechanical work during walking. *Sci. Rep.* **7**, 15404 (2017).

3. Zelik, K. E. & Honert, E. C. Ankle and foot power in gait analysis: Implications for science, technology and clinical assessment. *J. Biomech.* **75**, 1–12 (2018).
